# Cannabinoid type-1 receptor signaling in dopaminergic Engrailed-1 expressing neurons modulates motivation and depressive-like behavior

**DOI:** 10.3389/fnmol.2024.1379889

**Published:** 2024-04-04

**Authors:** Sarah Baddenhausen, Beat Lutz, Clementine Hofmann

**Affiliations:** ^1^Institute of Physiological Chemistry, University Medical Center of the Johannes Gutenberg University Mainz, Mainz, Germany; ^2^Leibniz Institute for Resilience Research (LIR), Mainz, Germany; ^3^Focus Program Translational Neuroscience, University Medical Center of the Johannes Gutenberg University Mainz, Mainz, Germany

**Keywords:** dopaminergic system, CB1 receptor, ventral tegmental area, motivation, depressive-like behavior

## Abstract

The endocannabinoid system comprises highly versatile signaling functions within the nervous system. It is reported to modulate the release of several neurotransmitters, consequently affecting the activity of neuronal circuits. Investigations have highlighted its roles in numerous processes, including appetite-stimulating characteristics, particularly for palatable food. Moreover, endocannabinoids are shown to fine-tune dopamine-signaled processes governing motivated behavior. Specifically, it has been demonstrated that excitatory and inhibitory inputs controlled by the cannabinoid type 1 receptor (CB1) regulate dopaminergic neurons in the mesocorticolimbic pathway. In the present study, we show that mesencephalic dopaminergic (mesDA) neurons in the ventral tegmental area (VTA) express CB1, and we investigated the consequences of specific deletion of CB1 in cells expressing the transcription factor Engrailed-1 (En1). To this end, we validated a new genetic mouse line EN1-CB1-KO, which displays a CB1 knockout in mesDA neurons beginning from their differentiation, as a tool to elucidate the functional contribution of CB1 in mesDA neurons. We revealed that EN1-CB1-KO mice display a significantly increased immobility time and shortened latency to the first immobility in the forced swim test of adult mice. Moreover, the maximal effort exerted to obtain access to chocolate-flavored pellets was significantly reduced under a progressive ratio schedule. In contrast, these mice do not differ in motor skills, anhedonia- or anxiety-like behavior compared to wild-type littermates. Taken together, these findings suggest a depressive-like or despair behavior in an inevitable situation and a lack of motivation to seek palatable food in EN1-CB1-KO mice, leading us to propose that CB1 plays an important role in the physiological functions of mesDA neurons. In particular, our data suggest that CB1 directly modifies the mesocorticolimbic pathway implicated in depressive-like/despair behavior and motivation. In contrast, the nigrostriatal pathway controlling voluntary movement seems to be unaffected.

## 1 Introduction

The endocannabinoid system (ECS) consists of cannabinoid type 1 and 2 receptors (CB1, CB2), endogenous ligands, namely endocannabinoids, and their synthesizing and degrading enzymes. The CB1 receptor is widely expressed in the adult brain. It is present in many different cell types and neurotransmitter systems, predominantly in presynaptic terminals of GABAergic and glutamatergic neurons, where its activation suppresses neurotransmitter release mediated by the release of endocannabinoids (eCBs) in the postsynapse (Piomelli, 2003; Kano et al., 2009; Lutz, 2020), but it functions also in glia cells, e.g., in astrocytes, to modulate synaptic and metabolic processes (Eraso-Pichot et al., 2023). Considering that cannabis is one of the most widely used illicit substances worldwide, leading to addiction, among other behavioral impacts (Volkow et al., 2017), together with the observation that cannabinoid receptor agonists modulate the activity of mesencephalic dopaminergic (mesDA) neurons (French et al., 1997; Gessa et al., 1998; Melis et al., 2012; Bloomfield et al., 2016; Frau et al., 2019), a detailed understanding of the ECS in the mesDA neurons is of high importance (Parsons and Hurd, 2015). MesDA neurons are the essential source of dopamine in the mammalian brain and are involved in the guidance of voluntary movements and in the regulation of emotion-influenced behavior. They are affected in neurological and psychiatric diseases, such as Parkinson’s disease, schizophrenia and depression, and are located in their majority in the ventroanterior ventral midbrain, i.e., in the ventral tegmental area (VTA), the substantia nigra pars compacta (SNc), as well as the retrorubal field (RRF) (Schultz, 2002; Smidt and Burbach, 2007). Neurons of the VTA project to the ventromedial striatum (including the nucleus accumbens, NAc), to the amygdala, hippocampus and cerebral cortex (e.g., prefrontal cortex, PFC) (Morales and Margolis, 2017; Baik, 2020). This so-called mesocorticolimbic pathway plays a critical role in emotional behavior and reward mechanisms. In particular, the projection to the NAc plays a prominent role in positive reinforcement and goal-directed behavior, resulting in reward acquisition (Hikida et al., 2010; Kravitz et al., 2012; Klawonn and Malenka, 2018), and there is mounting evidence for a pivotal role of the ECS in homeostatic and hedonic aspects of food intake (Matias et al., 2006; Kirkham, 2009; Ruiz de Azua and Lutz, 2019). The mesDA neurons of the SNc, located adjacent to the VTA in the ventral midbrain, are responsible for motor control. Axons arising from the SNc project to the dorsolateral striatum and the cerebral cortex. In humans, the failure of nigrostriatal dopamine neurotransmission and the loss of DA neurons in the SNc are prominent in Parkinson’s disease (PD) underlying motor dysfunctions (Kravitz et al., 2010; Kalia and Lang, 2015; Balestrino and Schapira, 2020). To study the putative role of CB1 also in the SNc we enclosed behavioral assays testing locomotion and motor behavior.

The CB1 receptor was shown to be expressed in reward and motivation regions, including the PFC and NAc (Marsicano and Lutz, 1999; Lane et al., 2012). However, CB1 protein or mRNA could not be detected or display only sparse levels in VTA and SNc (Herkenham et al., 1991; Matsuda et al., 1993). Nevertheless, dose-dependent activation of CB1 receptor enhances firing rate and burst activity of mesDA neurons in the VTA and promotes dopamine release in terminal regions such as the NAc (Tanda et al., 1997; Cheer et al., 2004). Thus, it is assumed so far that a direct effect of cannabinoids on DA cells seems unlikely. Instead, glutamatergic and GABAergic neurons in the VTA itself or in projecting areas, e.g., PFC, striatum, projecting to the VTA and SNc (Parsons and Hurd, 2015) and expressing CB1 (Matsuda et al., 1993; Marsicano and Lutz, 1999), can regulate dopamine neurotransmitter release (Melis et al., 2012).

Besides the role in adult brain, the ECS also participates in the regulation of various steps of neurogenesis during embryonic development, such as proliferation, migration, specification of developing neurons and axonal outgrowth (Williams et al., 2003; Berghuis et al., 2005; Aguado et al., 2006; Maccioni et al., 2008; Mulder et al., 2008; Maccarrone et al., 2014; Tortoriello et al., 2014). The complex spatio-temporal expression pattern of the ECS components during embryogenesis (CB1, CB2 and enzymes) reveals a dynamically regulated signaling system, supported by experiments using substances that agonize or antagonize the mode of functioning of ECS components in postnatal neurogenesis (Maccarrone et al., 2014). Furthermore, there is evidence for cross-talks with FGF-, BDNF-, and NCAM-triggered signaling pathways (Zhou et al., 2014). In the developing central nervous system (CNS), CB1 receptor starts in the midbrain/hindbrain border (MHB) to be expressed at embryonic day (E)10.5 (unpublished data), known as the isthmus or isthmic organizer, which is necessary and sufficient for the development and formation of mesencephalic and metencephalic identities (Wurst and Bally-Cuif, 2001). Furthermore, the isthmus is essential for the specification of the midbrain dopaminergic neurons. The region in which mesDA neurons are born is designated by the isthmus, which produces fibroblast growth factor 8 (FGF8), and which, together with Sonic hedgehog (SHH), secreted from the ventrally located floor plate, specifies the region of prospective DA neurons at the intersection region of the two signals. DA neuron precursors are specified and located at the rostral side of the isthmus (Hynes et al., 1995; Hynes and Rosenthal, 1999; Smidt and Burbach, 2007; Garritsen et al., 2023).

During development, CB1 is also expressed in the isthmus and its expression domain is encompassed by the Engrailed-1 (En1) positive domain (unpublished data). The homeobox transcription factor En1 in the isthmus controls, together with its paralogue En2, the developmental fate of mesDA neurons beginning from their generation (Simon et al., 2001; Albéri et al., 2004). To elucidate the role of CB1 in developing and mature mesDA neurons, we have generated a new conditional mouse line, bearing a deletion of the CB1 gene in En1 expressing cells, thus leading to an inactivation of CB1 in the “precursor domain” of mesDA neurons. En1 is expressed first at the one-somite stage in the anterior neural folds and continues expression to E8 during embryogenesis (Wurst et al., 1994). Its early developmental role arises from a broad band of expression rostral and caudal to the mid-hindbrain border at E9. At around E11, En1 is highly expressed by all differentiating mesDA neurons generated in the isthmus (Davis and Joyner, 1988). Although En1 and En2 are cell-autonomously required for proper development, i.e., the survival and maintenance of mesDA neurons in a gene dose-dependent manner, they are not necessary for their induction and specification (Simon et al., 2001). The expression of En1 is maintained throughout adulthood and essentially in all mesDA neurons in the VTA and SNc (Liu and Joyner, 2001; Simon et al., 2001; Albéri et al., 2004). En genes also prevent apoptosis of mesDA neurons, and mutant mice null for En1 and En2 exhibit a complete absence of the tectum and cerebellum (Liu and Joyner, 2001; Simon et al., 2001). Nevertheless, mesDA neurons become postmitotic and acquire a neurotransmitter phenotype by expressing tyrosine hydroxylase (TH), the rate-limiting enzyme of dopamine synthesis. However, at birth, the entire population of mesDA neurons is disappeared in En1/En2 double mutants (Albéri et al., 2004). Furthermore, En1-null mutant mice die at birth and exhibit an almost complete deletion of the midbrain and cerebellum owing to tissue loss by E9.5 (Wurst et al., 1994). These and other loss and gain of function experiments firmly established that En1 and En2 are survival factors for DA neurons *in vivo*. It was also reported that in mice lacking only one En1 allele (e.g., En1LacZ^+/−^ mice; Sonnier et al., 2007) crossed in wild-type Swiss mice (En1^+/−^), the number of DA neurons is reduced progressively with age from 8 and 24 weeks postnatal to remain stable at 38 and 23% reduction in the SNc and VTA, respectively. Neuronal degeneration is concomitant with a 37% decrease in striatal dopamine. Furthermore, En1^+/−^ mice exhibit, e.g., decreased spontaneous locomotor activity and an anhedonic-like behavior compared with their wild-type littermates (Sonnier et al., 2007; Nordström et al., 2015). Based on these findings, we included in our analyses also En1^Cre/+^ mice maintained on a C57BL/6 J wild-type background (EN1-CRE), which was essential for our analysis to differentiate a potential effect due to one impaired En1 allele from a potential effect of missing CB1.

In the present study, we generated a mutant mouse line in which CB1 receptor is deleted in En1-expressing cells (EN1-CB1-KO). We found that in wild-type mice, CB1 is expressed in a subset of mesDA neurons within the VTA, while in EN1-CB1-KO mice, CB1 expression is lost in mesDA neurons. Mutant mice exhibit an increased despair behavior in the forced swim test and display a decreased motivation in reward-directed behavior for palatable food in the progressive ratio test. Importantly, these phenotypes are caused by the deletion of CB1 and are not attributed to the absence of one En1 allele, as no differences were observed between EN1-CRE and EN1-WT littermate controls. Furthermore, in contrast to En1^lacZ/+^ haploinsufficient mice, our EN1-CRE mice do not display impaired motor skills. Moreover, stereological counts of TH-positive cells revealed that neither in EN1-CB1-KO nor EN1-CRE mice, the number of mesDA neurons in the VTA and SNc was altered compared to their respective control littermates. Our findings presented here provide new insights into the involvement of the ECS in the mesocorticolimbic system concerning depressive-like behavior and motivation for palatable food.

## 2 Materials and methods

### 2.1 Animals

#### 2.1.1 EN1-CB1-KO mouse line

Experiments were carried out with male mice of the EN1-CB1-KO line. Since mice were consecutively subjected to the different behavioral tests, the exact age is indicated in the respective Result section and is summarized below. For the generation of this new mouse line, En1^Cki^ mice were crossed with CB1^f/f^ mice (on C57BL/6 J background), in which the CB1 gene is flanked by two loxP sites (Marsicano et al., 2003). The EN1-CRE knock-in (En1^Cki^) line was generated by Kimmel et al. (2000) using an En1 driven knock-in vector (Hanks et al., 1995), thereby inactivating the endogenous En1 gene, and was provided by Wolfgang Wurst (Helmholtz Centre, Munich). EN1-CB1-KO mutant mice harbor one Cre recombinase allele within the endogenous En1 gene and two alleles of CB1^f/f^, control mice carry the En1 wildtype allele without Cre and both alleles of CB1^f/f^. Mutant mice were herein referred to as EN1-CB1-KO and control littermates as EN1-CB1-WT.

#### 2.1.2 EN1-CRE mouse line

Furthermore, the EN1-CRE mouse line was used as an additional control to ensure that a potential effect is due to the En1-specific deletion of CB1 and not caused by the functional impairment of one En1 allele. The EN1-CRE line was maintained by crossing En1^Cki^ mice with C57BL/6 J. Mutant mice were referred to as EN1-CRE, control littermates as EN1-WT.

In both lines, siblings served as direct controls. Adult male mice were housed individually in controlled laboratory conditions during behavioral studies, with the temperature maintained at 21 ± 1°C and humidity at 55 ± 1%. Unless otherwise described, due to organizational restrictions, mice were tested during the light phase (lights on at 7 a.m. and off at 7 p.m.), and the animals were used in a rotational principle, i.e., every experimental day was started with different mice to avoid possible circadian influence on behavioral responses. Mice had access to food and water *ad libitum*. Experimenters were blind to the genotype of mice. Experiments were carried out in accordance with the Council Directive 2010/63EU of the European Parliament and the Council of September 22, 2010, on the protection of animals used for scientific purposes and approved by the local Ethical Committee on animal care and use of Rhineland-Palatinate, Germany (Landesuntersuchungsamt Koblenz, permit number 23177-07/G 16–1-084). An important point to be considered is that experimental mice were housed individually, which bears the risk of social isolation. In our study, though, the primary outcome was the comparison between two genetically different mouse lines. Hereby single versus group housing might influence whether or not we might see a genotype difference. Thus, we can exclude false positive results (i.e., genotype differences) in our experimental design. Furthermore, only male mice were tested. We are aware of the fact that both male and female mice should be tested in evaluating behavior and that especially in anxiety- and depressive-like behavior the difference between both may become evident, i.e., females might be more sensitive. Unfortunately, due to constraints in equipment and work resources, we were not able to perform the experiments also in females.

#### 2.1.3 Order of tests presented to the mice and age of animals used

The performed tests are listed in the same order as they were presented to the mice. We were using for both lines two batches each: Cohort #2 mice (starting at 12–14 weeks of age) were taken for light/dark test (LD) and forced swim test (FST); after 1 week break they passed through the progressive ratio (PR) test with food restriction. Cohort #3 mice (starting at 12–16 weeks of age) went through Open field (OF), Rotarod, Sucrose preference test and, at 30–33 weeks of age, again OF, Rotarod, and (for the first time) FST; after a 2 months break, PR without food restriction was performed.

#### 2.1.4 Genotyping

Genotyping was performed by polymerase chain reaction (PCR) from tail or ear genomic DNA using the forward primer 5′ –GTGCCT TCGCTG AGGCTTC- 3′ (en1 fwd) and the reverse primer 5′ –ACCCTG ATCCTG GCAATT TCGGC- 3′ (en1 rev; which is located within the *Cre* sequence) for the detection of the En1^Cki^ locus, yielding a 600 bp band, for the wild-type alleles no band. The floxed *CB1* receptor allele was detected using the forward primer 5′ –GCTGTC TCTGGT CCTCTT AAA- 3′ (G50) and reverse primer 5′ –GGTGTC ACCTCT GAAAAC AGA- 3′ (G51). Using these primers, wild-type alleles give an amplicon of 413 bp, whereas floxed alleles a 493 bp amplicon. The evaluation of both loci was done simultaneously in one PCR reaction with the following conditions: 1× 94°C for 3 min; 27x (94°C for 1 min; 57°C for 30 s; 72°C for 45 s); 1× 72°C for 3 min.

### 2.2 Immunofluorescent staining

Mice were anesthetized with pentobarbital, transcardially washed, and perfused with PBS (phosphate buffered saline: 136 mM NaCl, 2.7 mM KCl, 10 mM Na_2_PO_4_; pH 7.4) and 4% paraformaldehyde (PFA) in PBS solution, respectively. The isolated brains were post-fixed afterwards in 4% PFA/PBS overnight at 4°C. After incubation in 30% sucrose/PBS solution for 48 h, brains were frozen on dry ice and stored at −20°C until use. Next, 30-μm thick coronal brain slices were cut on a cryostat Microtome HM560 (Microm, Walldorf, Germany) and kept at 4°C in cryoprotection solution (25% glycerin, 25% ethylene glycol, 50% PBS) until use. Free-floating sections were rinsed from cryoprotection solution with PBS containing 0.2% Triton X-100 (3051.2, Roth, Germany) (PBS-TX) for 10 min, then pre-incubated for 15 min in blocking solution (4% goat serum in PBS) before incubating with the respective primary antibodies overnight at 4°C (mouse anti-TH: F-11; Santa Cruz Biotechnology Inc., CA, United States; 1:200; rabbit anti-NeuN: EPR12763, Abcam, Cambridge, United Kingdom). On the next day, the sections were washed three times for 10 min with PBS-TX and then incubated for 1 h with the appropriate Alexa488- or Alexa546-conjugated goat IgG (goat anti-rabbit Alexa-488 and donkey anti-mouse Alexa-546, Invitrogen, Carlsbad, CA, United States; 1:1000). Brain slices were incubated with DAPI (4’,6-Diamidino-2-Phenylindole, Dihydrochloride, D1306 Invitrogen, CA diluted 1:5000 in PBS) for 5 min to visualize cell nuclei. After a final washing step in PBS, sections were mounted with Mowiol 4–88 mounting medium (Roth, Karlsruhe, Germany) onto slides and dried overnight. Fluorescence labeling was visualized using a Leica DM5500 fluorescence microscope (Leica microsystems, Wetzlar, Germany). Images were captured by using the 20x objective.

### 2.3 Stereological counts of mesDA neurons

Manual quantification of mesDA neurons in the VTA and SNc was performed by counting every sixth section of TH-positive cells between −2.54 mm and − 3.26 mm posterior to bregma (30-μm-slices x 4 analyzed sections x every 6^th^ section = 720 μm) along the anterior–posterior axis for each animal (EN1-CB1-KO *n* = 4; EN1-CB1-WT *n* = 4 and EN1-CRE *n* = 5; EN1-WT *n* = 4). Numbers were then extrapolated for the whole region by multiplying by 6, averaged for every genotype, and compared between groups.

The total number of neurons was determined by counting NeuN-positive cells in this 720 μm analyzed region (EN1-CB1-KO *n* = 3; EN1-CB1-WT *n* = 3 and EN1-CRE *n* = 2; EN1-WT *n* = 3). VTA and SNc were identified according to anatomical landmarks by the Paxinos mouse brain atlas (2008). For stereological cell counts, the LASX software was applied. Statistical analysis was conducted by a two-tailed unpaired Student’s *t*-test.

### 2.4 Fluorescent *In situ* hybridization

Double fluorescence *in situ* hybridization tests were carried out on coronal slices. To identify CB1/TH double-positive neurons, VTA and SNc were analyzed using fluorescein isothiocyanate (FITC) labeled riboprobes for CB1 and digoxigenin (DIG) labeled riboprobes for TH, and vice versa, when Gad65 (FITC labeled) was used to detect co-expressed CB1 (DIG labeled). VTA and SNc were analyzed in three EN1-CB1-KO mice and three EN1-CB1-WT littermates between −2.54 mm and − 3.26 mm posterior to the bregma. Adult mice were sacrificed by decapitation under deep isoflurane anesthesia. Brains were isolated, snap-frozen on dry ice, and stored at −80°C. Tissue Freezing Medium (Leica Biosystems) was used to mount the brains for sectioning, and 18 μm-thick coronal slices were cut from rostral on a cryostat Leica CM3050 S, dried on a 42°C heating plate, and stored at −20°C until use.

Riboprobes labeled with DIG and FITC were used. The DNA templates for the CB1 and TH probes were originally synthesized by reverse transcription polymerase chain reaction (RT-PCR) from whole mouse brain cDNA, as previously described (Marsicano and Lutz, 1999; Guimera et al., 2006). A list of the GenBank accession number, primer sequences, and probe length is reported there.

*In vitro* transcription of all three probes was performed for 3 h at 37°C in 20 μL of the linearized plasmid containing 2 μg of CB1, TH or Gad65 gene inserts. The following restriction enzymes were used for linearization and RNA polymerases: CB1 antisense: BamHI, T3; CB1 sense: EcoRI, T7; TH antisense: SacII, Sp6; TH sense: NotI, T7; Gad65 antisense: Bam HI, T3; Gad65 sense: Eco RI, T7. Pretreatment, hybridization, and imaging of the fluorescent *in situ* hybridization protocol were carried out as previously described (Zimmermann et al., 2018). The TH riboprobe was labeled with digoxigenin and used in the hybridization reaction over night at a final concentration of 80 ng/mL hybridization mix, while the CB1 riboprobe was labeled with FITC or DIG and applied onto the sections at a final concentration of 800 ng/mL. Gad65 was labeled with FITC and used at a final concentration of 300 ng/mL. Positive signals were detected by incubating hybridized sections with α-Digoxigenin-AP or α-Fluorescein-AP (Roche Diagnostics), followed by TSA Plus Cyanine and TSA Fluorescein amplification solution, respectively (Perkin Elmer/Akoya Biosciences).

### 2.5 Behavioral tests

#### 2.5.1 Open field test

Mice were placed in one corner of an illuminated (90 lux) white box (40 × 40 × 40 cm^3^) and allowed to explore the open field for 10 min. After each trial, the open field was cleaned with 70% ethanol. Distance traveled, velocity, and time in the center was tracked by EthoVision XT 15 software.

#### 2.5.2 Light/dark test

The light/dark (LD) test was performed in a box divided into an open, white, brightly illuminated (100 lux at entry site) compartment (40 × 27 × 40 cm^3^) and a closed, black, dark compartment (40 × 13 × 40 cm^3^). Mice were placed in the dark compartment and then allowed to explore the entire box for 5 min. After each trial, the box was cleaned with 70% ethanol. Time in the lit zone was tracked by EthoVision XT 15. The experimenter, blind to the genotype, manually scored the risk assessments and number of entries to the light. Furthermore, the latency to the first entry and the time spent in the lit zone were used as parameters to determine aversive behavior.

#### 2.5.3 Forced swim test

Each mouse was placed in a round glass beaker (20 cm high; 4,500 mL volume) filled with tap water (25 ± 0.5°C). Mice were video recorded for 6 min. The behavior of the last 4 min was analyzed manually and blind to the genotype. The immobility time was defined as the duration of a mouse floating in the water without struggling and making only small movements to keep its head above the water.

#### 2.5.4 Accelerating rotarod

The rotarod apparatus (Ugo Basile, Comerio, Italy) measures motor coordination and balance. Mice were placed on the rod at an accelerating speed ranging gradually from 4 to 40 rpm over 5 min. They were trained in three trials per day for three consecutive days. The average per day of the 3 days was calculated for young (12–13 weeks of age) and older (30–31 weeks of age) adult mice to analyze both their motor coordination and ability to learn not to fall off the rotarod.

#### 2.5.5 Sucrose preference test

The test was conducted in the home cage equipped with two drinking bottles containing tap water and a 1 or 2% sucrose solution. Mice were habituated for 12 h to the presence of the two bottles filled only with water. Following this habituation, mice could choose either to drink sucrose solution or water for 3 days. The positions of the bottles were switched every 24 h to reduce any side preference. Water and sucrose solution intake was measured daily after 24 h, and sucrose preference was calculated as a percentage of the volume of sucrose intake over the total volume of fluid intake.

#### 2.5.6 Progressive ratio paradigm with food restriction

(I) Operant self-administration apparatus: Mouse operant chambers (ENV-022MD, Med Associates, Georgia, VT, United States) were used for operant responding maintained by chocolate-flavored pellets. Chambers were equipped with a touch-sensitive thin-film transistor (TFT) display covered with a black metal partition containing a response hole (ø = 1.5 cm), allowing the presentation of the visual stimuli (light). Touching the light screen resulted in a pellet delivery on the opposite side of the chamber into a food tray (magazine). It was paired with a stimulus light (associated cue) located above the magazine, signaling the delivery of the chocolate-flavored pellet. A food dispenser permitted the delivery of the pellets when required. The floor of the chambers was a grid floor. The chambers were placed in sound- and light-attenuated boxes. The experiment was controlled by the K-Limbic software (Conclusive Marketing Ltd., Herts, United Kingdom).

(II) Food pellets: After touch responding, animals received highly palatable 20 mg chocolate-flavored pellets (BioServ, Flemington, NJ, United States). The pellets had a caloric value of 3.60 kcal/g containing 18.4% protein, 5.5% fat, 4.6% fiber, 6.5% ash, and 59.1% carbohydrate and addition of chocolate flavor.

(III) Food restriction: One week before the start of the self-administration sessions, mice were placed on a schedule of controlled feeding and maintained at 85% of their free-feeding body weight for the whole duration of the experiment. Thereby weight of each animal was determined daily before every session. Water was available *ad libitum*.

(IV) Self administration session: Mice were tested on seven consecutive days per week. Every mouse underwent the following sequence of phases:

(1) Habituation

First, mice were familiarized with the chocolate-flavored pellets in their home cage overnight, followed by acclimation to the operant chambers on four consecutive days for 30 min/day. In this phase, pellets were available *ad libitum* in the magazine with an inter-trial of 15 s. A new trial was initiated after the disruption of an infrared light beam by the pellet collection.

(2) Fixed ratio

The daily self-administration sessions lasted 30 min. Pellets were delivered after a touch response paired with the associated cue light. A time-out period of 10 s was established after every pellet delivery, where the cue light was off, and no reinforcer was provided after responding on the touch screen. In the operant conditioning sessions, mice were first trained under a fixed ratio (FR) 1 schedule of reinforcement where one touch response resulted in one pellet delivery. Subsequently, mice had to pass an increased FR2 training where two touch responses resulted in one pellet delivery and an FR4 training where four touch responses resulted in one delivery. The criteria for completion of the operant FR training were acquired when the mice collected at least 50 pellets within one session. Thus, mice were led to the following FR schedule individually when criteria were finally fulfilled. Therefore, animals were not synchronized.

(3) Progressive ratio

The progressive ratio (PR) schedule of reinforcement was used to evaluate the motivation for chocolate-flavored pellets. The response required to earn one single pellet escalated according to the following series: 2, 4, 6, 8, 11, 14, 17, 21, 25, 29, 34, 39, 44, 50, 56, 62, 69, 76, 83, 91, 99, 107, 116, 125, 134, 144, 154, 164, and 174. The session was completed after 1.5 h or 29 collected pellets. The maximal number of responses mice had performed to obtain one pellet was defined as the breaking point (BP).

Every session was signaled by turning on a house light placed on the chamber’s ceiling and switched on for the whole session. Afterwards, mice were returned to their home cages, and the chambers were cleaned to prevent the presence of the odor of the previous mouse.

To ensure that the decreased motivation is independent of genotype-specific hunger levels, we furthermore measured the amount of food intake in food-deprived mice.

#### 2.5.7 Progressive ratio paradigm without food restriction

(I) Operant self-administration apparatus: The same apparatus described as in the PR paradigm with food restriction was used. The only difference to the setup described above was that there was no other light on during the entire experiment but the stimulus light (associated cue) to maintain the dark phase in which mice were tested (details see below).

(II) Food pellets: The same food pellets as described in the PR paradigm with food restriction were used.

(III) Self-administration session: The mice were given approximately 4.5–5 g of chow per mouse throughout the experiment as it was necessary to maintain a body weight of 95% of their initial *ad libitum* body weight. Any remaining food was removed from the cage 1 h before behavioral testing. Water was available *ad libitum*. The mice were tested 7 days per week during the dark phase (lights off at 7.30 a.m. and on at 7:30 p.m.). Every very mouse underwent the following sequence of phases:

(1) Habituation

Mice were habituated to an inversed light/dark cycle for 12 days to ensure a complete adjustment to the new circadian rhythm. During this period, they were handled daily to habituate them to the room. For food habituation, chocolate-flavored food pellets were available in the home cages for two nights before the experiment started.

(2) Fixed ratio

Training sessions of operant responding were performed according to protocols previously described (Martín-García et al., 2011). Briefly, daily self-administration sessions maintained by chocolate-flavored pellets lasted 1 h per day. In the operant conditioning sessions, mice were under an FR1 schedule of reinforcement (one touch response resulted in one pellet delivery) followed by an increased FR5 (five touch responses resulted in one pellet delivery) for the rest of the sessions. Following criteria were identified before the start of the experiment for completing each training phase: (1) mice maintained a stable responding with less than 20% deviation from the mean of the total number of reinforcers earned in three consecutive sessions (80% of stability), (2) at least 75% responding on the active hole, and (3) a minimum of 10 reinforcers per session. Hence, all animals needed 13 days (6 days for FR1 and 7 days for FR5) to fulfill these criteria and thus were synchronized.

(3) Progressive ratio

The PR schedule of reinforcement was used to evaluate the motivation for the chocolate-flavored pellets. The response required to earn one single pellet escalated according to the following series: 2, 4, 6, 8, 11, 14, 17, 21, 25, 29, 34, 39, 44, 50, 56, 62, 69, 76, 83, 91, 99, 107, 116, 125, 134, 144, 154, 164, 175, 186, and so on. The maximal number of responses the mouse performs to achieve the last pellet is referred to as the breaking point (BP). The maximum duration of the PR session was 4 h or until mice did not respond within 1 h.

(IV) Extinction: Extinction sessions were performed in accordance with the procedure described before (Chung et al., 2015). In brief, experimental conditions were similar to the self-administration sessions except that the chocolate-flavored pellets were not available. Mice were given 1-h daily sessions (7 days/week) until reaching the extinction criterion. The extinction criterion was achieved when during three consecutive sessions, mice made a mean number of touch responses of less than 30% of the responses obtained during the mean of the three consecutive days taken to achieve the acquisition criteria of chocolate self-administration training in FR5.

All sessions were conducted in a dark operant chamber. After each session, mice were returned to their home cages, and the chambers were cleaned to prevent the presence of the odor of the previous mouse.

### 2.6 Statistical analysis

Data are represented as the mean ± standard error of the mean (SEM). Graphs and statistics were generated by GraphPad Prism 7 (GraphPad Software Inc., La Jolla, CA, United States) and IBM SPSS Statistics 23 v software (IBM Corporation, Armonk, NY, United States). Unless otherwise described, data were analyzed using a two-tailed unpaired Student’s *t*-test. Results were considered as significant at *p* < 0.05.

## 3 Results

### 3.1 The CB1 receptor is expressed in mesencephalic dopaminergic neurons in wild-type mice and lost in EN1-CB1-KO mutants

The present study investigated whether CB1 deficiency in mesDA neurons beginning from E10.5, when precursor mesDA neurons start to be determined in the isthmic organizer (Smits et al., 2006), affects adult behaviors that are controlled by mesocorticolimbic and nigrostriatal pathways.

**Figure 1:**
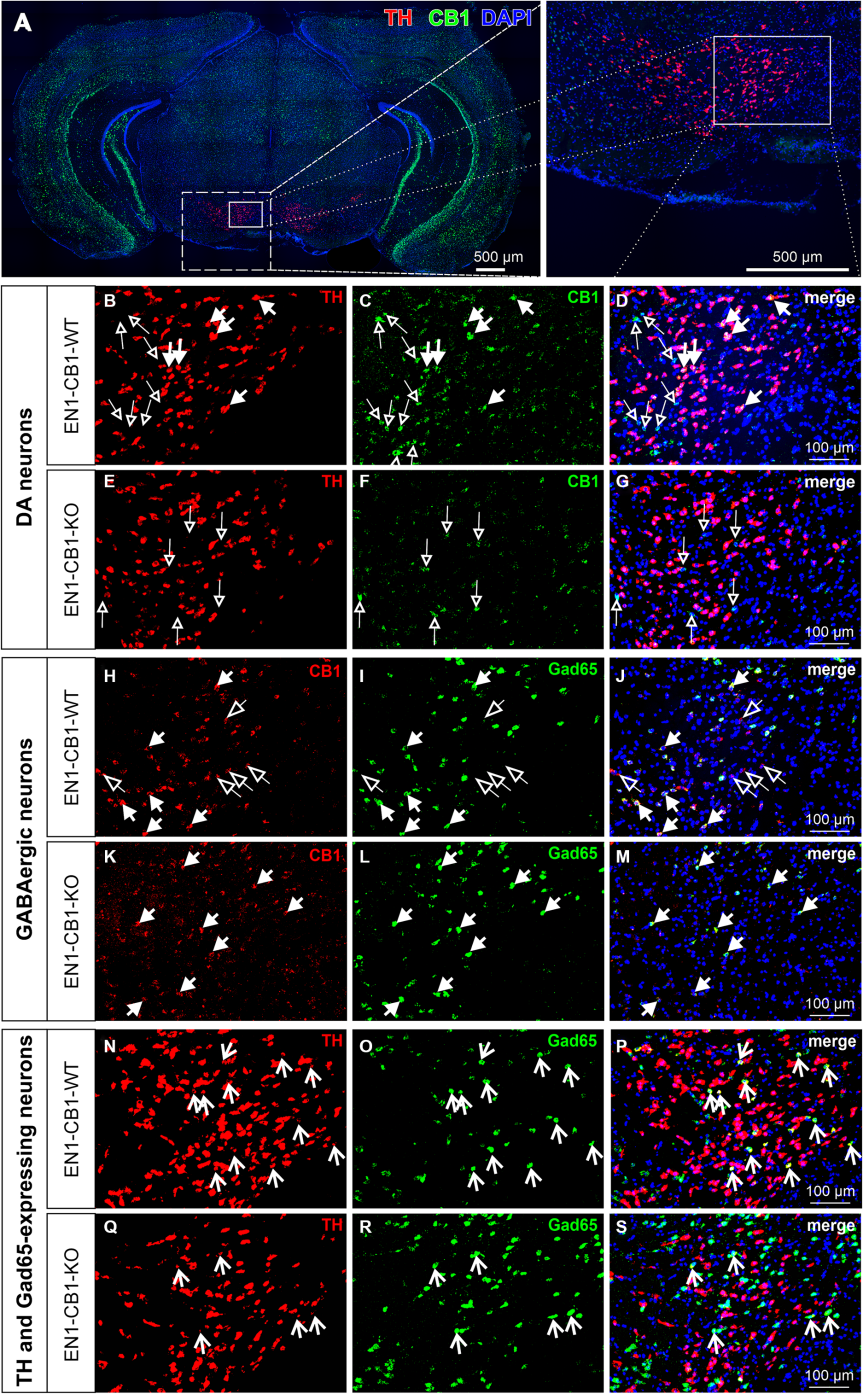
Co-expression of TH and CB1 mRNA in the VTA of EN1-CB1-WT mice and lack of CB1 expression in TH^+^ neurons in EN1-CB1-KO mutants. Representative micrographs of double fluorescence *in situ* hybridization experiments, performed on coronal midbrain sections of adult mice with a DIG- and a FITC-labeled riboprobe to detect mRNAs of TH and CB1, respectively **(A)**. High magnifications of medial to lateral part of VTA indicated in **(A)**: **(B–D)**, EN1-CB1-WT, **(E–G)**, EN1-CB1-KO. TH-expressing cells (red, **B,E**), CB1 transcripts (green, **C,F**), and merged images (also with DAPI nuclear stain, blue,) of **(B,C,E,F)**, respectively **(D,G)**. Filled arrows indicate cells co-expressing TH and CB1 in EN1-CB1-WT **(B–D)**, which are not present in mutant littermates **(E–G)**. CB1 is also expressed in TH-negative cells (empty arrows) both in EN1-CB1-WT **(C,D)** and KO mice **(F,G)**. However, a number of TH-positive mesDA neurons do not express CB1 (**B**, cells not marked) or show weak signals. **(H–M)** *In situ* hybridization of consecutive sections treated with a DIG- and FITC-labeled riboprobe to detect transcripts of CB1 and Gad65, respectively. CB1 is expressed in both, GABAergic interneurons (Gad65-positive; filled arrows) and Gad65-negative cells (empty arrows) of EN1-CB1-WT mice **(H–J)**. In EN1-CB1-KO mutants, CB1 is almost exclusively expressed in Gad65-expressing cells **(K–M)**. Moreover, in both genotypes a few cells express both, TH and Gad65, revealing a co-transmitter phenotype of some VTA neurons **(N–S)**. Scale bars: **(A)** 500 μm; **(B–S)** 100 μm.

First, we investigated whether and to what extent the CB1 receptor is expressed in mesDA neurons in adulthood. The double fluorescent *in situ* hybridization (FISH) method was carried out on coronal midbrain sections of EN1-CB1-KO mice and EN1-CB1-WT control littermates to describe the presence of CB1 and TH mRNA in adult mesDA neurons. DA neurons in the VTA and SNc were identified by TH-expression and using the atlas of Paxinos and Franklin (2008). While the dorsal tegmentum of the midbrain and the hippocampus exhibited higher CB1 fluorescence signals, the ventral tegmentum contains densely packed, mostly low CB1 expressing cells (Figures 1A,C). In EN1-CB1-WT mice, intense TH signal is detected (Figures 1A,B), and CB1 co-expressing with TH was identified in a subset of VTA cells, exhibiting weaker CB1 fluorescence signals (Figures 1B–D, filled arrows). Most co-expressing neurons were identified in the lateral VTA, a DA (TH^+^) cell body rich zone, comprising the parabrachial pigmented nucleus and paranigral nucleus [according to Baik (2020) and Garritsen et al. (2023)]. No co-expression was detected in the SNc (not shown). In mesDA neurons of EN1-CB1-KO mutant mice, CB1 mRNA expression is lost in TH-positive cells (Figures 1E–G). Only CB1 expression in non-TH-positive cells was still detected (Figure 1F, empty arrows). High CB1 mRNA expressing cells within the VTA do not express TH (Figures 1B–D, empty arrows) being most likely GABAergic neurons as the VTA consists besides of DA neurons (65%) mainly of GABAergic (33%) neurons (Nair-Roberts et al., 2008; Morales and Root, 2014), which are known to express CB1 (Hill et al., 2007; Busquets-Garcia et al., 2018). Indeed, by an additional double FISH carried out on parallel sections with a CB1-DIG- and glutamic acid decarboxylase 65 (Gad65)-FITC-labeled riboprobe, we identified strong CB1 signals in GABAergic interneurons of EN1-CB1-WT mice (Figures 1H–J, filled arrows), while weak CB1 signals do not exhibit Gad65 signals (Figures 1H–J, empty arrows). In EN1-CB1-KO mice, most of the CB1 signal was attributable to a Gad65-expressing GABAergic neuron (Figures 1K–M, filled arrows). Moreover, the VTA was analyzed regarding co-expression of TH and Gad65 (Figures 1N–S). Indeed, a few cells are expressing both, TH and Gad65 (see arrows), revealing a cotransmitter phenotype of some VTA neurons.

### 3.2 EN1-CB1-KO and EN1-CRE mice do not show a decline in the number of mesencephalic dopaminergic neurons

**Figure 2:**
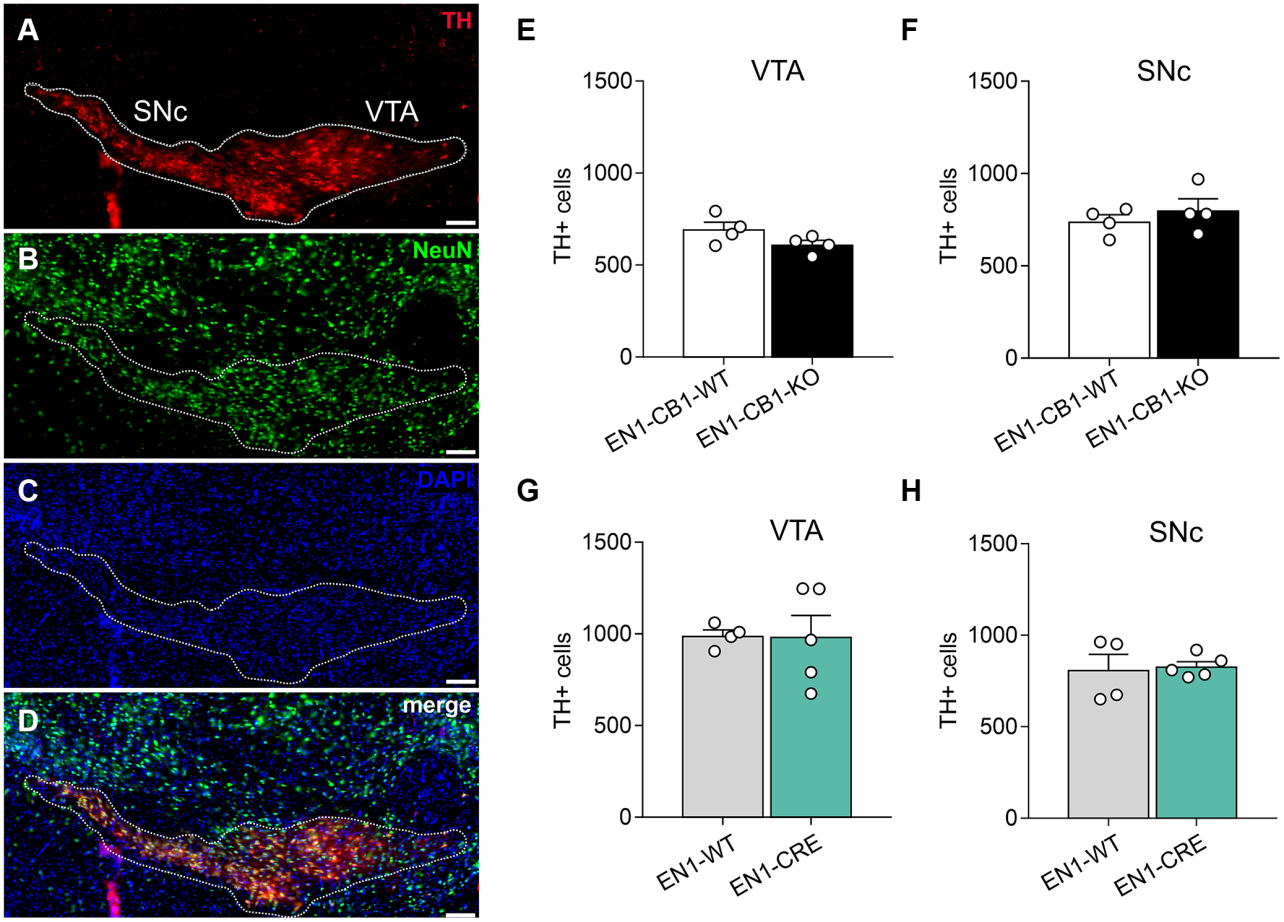
Quantifications of mesDA neurons of EN1-CB1-KO and EN1-CRE mouse lines. **(A–D)** Representative coronal section of the VTA and SNc of a 42-weeks-old wild-type mouse analyzed by immunohistochemistry. DA neurons were stained for TH **(A)**, the total number of neurons by NeuN **(B)**, cell nuclei by DAPI **(C)**, and a merge of TH, NeuN and DAPI is shown **(D)**. The white contour shows the borders of VTA and SNc. **(E–H)** Stereological cell counts in the VTA and SNc of EN1-CB1-KO **(E,F)** and EN1-CRE mice **(G,H)** in comparison to their respective WT controls. Data represent means ± SEM for each group (*n* = 4–5). SNc, substantia nigra pars compacta; TH, tyrosine hydroxylase; VTA, ventral tegmental area. Scale bar: 100 μm.

Next, we needed to exclude a potential haploid effect of the En1^Cki/+^ locus on a C57BL/6 J wild-type background (EN1-CRE), and we used C57BL/6 J littermates (EN1-WT) as controls. Sonnier et al. (2007) and Nordström et al. (2015) showed that heterozygous En1^+/−^ mice are born with a normal number of mesDA neurons, but display an age-dependent progressive cell loss reaching by 24 weeks of age a 38 and 23% reduction in the SNc and VTA, respectively. In both publications, heterozygous En1 mice with a lacZ insertion in the En1 locus were used (Hanks et al., 1995). In contrast, our study analyzed the heterozygous En1^Cki/+^ mouse line with a Cre recombinase sequence insertion, replacing the first 111 amino acids of one En1 allele (Kimmel et al., 2000), and thereby possibly also compromising endogenous En1 gene function in the heterozygous state. For this reason, we analyzed 42-weeks-old mice using immunohistochemistry to determine whether En1^Cki/+^ mice showed a loss of mesDA neurons (Figure 2). We compared not only EN1-CB1-KO mice and EN1-CB1-WT littermate controls but also EN1-CRE mice and EN1-WT littermates. By stereological counts of dopaminergic cells positive for TH, we evaluated the number of mesDA neurons in the VTA and SNc (Figure 2A). Moreover, we quantified the total number of neurons on the same sections by using the pan-neuronal nuclear marker NeuN (Figure 2B). Stereological counts revealed no genotype differences in the total number of TH-positive cells neither in the VTA nor in the SNc of both mouse lines (numbers shown in Figures 2E–H; *p* > 0.05, two-tailed unpaired Student’s *t*-test). Furthermore, the total number of NeuN-positive neurons was not altered either in the EN1-CB1-KO or EN1-CRE mice as compared to their respective controls (EN1-CB1-KO: 1725 ± 83.82; EN1-CB1-WT: 1861 ± 74.24, *p* = 0.2694; EN1-CRE: 1898 ± 144.5; EN1-WT: 1686 ± 58.09, *p* = 0.2049; two-tailed unpaired Student’s *t*-test). Hence, we conclude that neither the heterozygosity of the En1 gene in the here used En1^Cki/+^ mouse line nor the ablation of CB1 causes degeneration of mesDA neurons.

### 3.3 Behavioral analysis reveals a role for CB1 receptor in motivation and depressive-like behavior, whereas locomotion is not affected

#### 3.3.1 Locomotion, anxiety-like behavior, and sensorimotor learning

We next investigated the effect of CB1 absence in DA precursors and differentiated (adult) DA neurons on the adult behavioral phenotype of EN1-CB1-KO mice. First, we performed locomotion tests, since mesDA neurons from the SNc projecting to the dorsal striatum are necessary for voluntary movements. We conducted locomotion tests with mice of different age: 12–13 weeks old (young adults) and 30–31 weeks of age (old adults), to address locomotor skills and asked whether a decline in motor performance with age is detectable.

**Figure 3:**
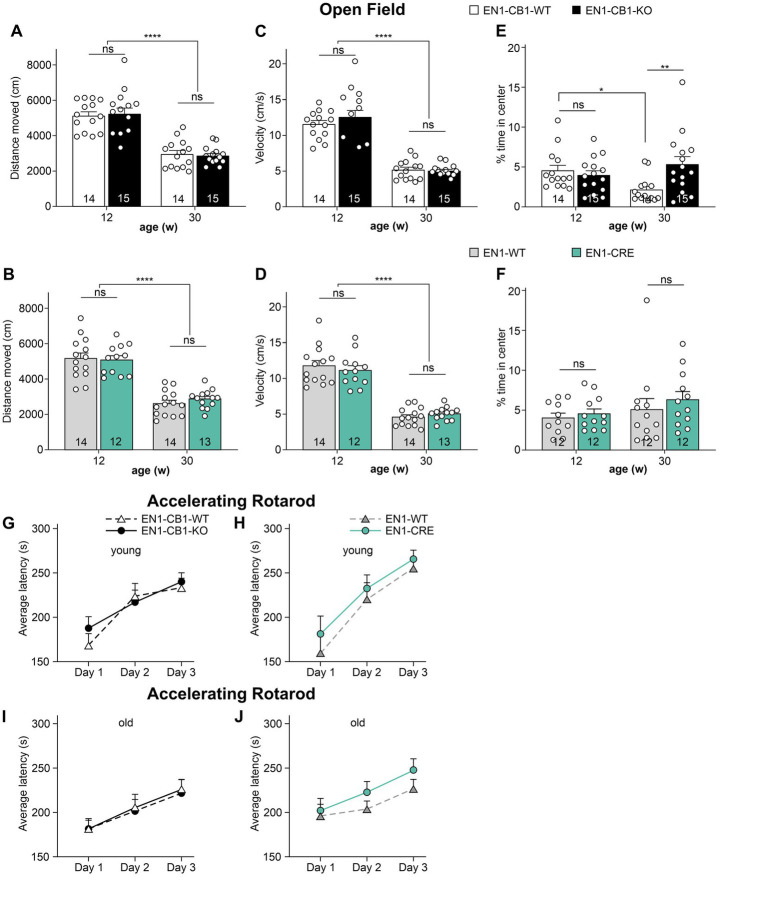
Analysis of locomotion, anxiety-like behavior **(A–F)** and motor coordination **(G–J)** in EN1-CB1-KO and EN1-CRE mouse lines. 12–13 and 30–31 weeks old EN1-CB1-KO and EN1-CRE mice compared to their respective control mice, expressed as the mean ± SEM of distance moved **(A,B)**, velocity **(C,D)**, and time spent in the center zone (%) **(E,F)**. EN1-CB1-KO and EN1-CRE mice do not show differences compared to littermate controls in the parameters distance moved and velocity. However, older EN1-CB1-KO animals seemed to stay for longer time in center as compared to their controls, suggesting a decreased anxiety-like behavior **(E)**. However, comparing the 12 weeks old EN1-CB1-WT to the 30 weeks old EN1-CB1-WT controls **(E)**, old WT mice stay significantly shorter in the center than young EN1-CB1-WT mice suggesting an increased anxiety-like behavior with the age, a phenotype which old EN1-CB1-KO mice do not show. A decrease in moved distance and velocity between young and old adults was observed in both mouse lines but without differences between the genotypes. Rotarod test **(G–J)**. Analysis of accelerating rotarod test: Performances of 13 and 31 weeks old EN1-CB1-KO and EN1-CRE mice and their control littermates expressed as the average ± SEM of latency (s) to fall off the rod (mean of three trials). Mice improved in motor coordination, balance, and sensorimotor learning without genotype differences both in young and old adults. Repeated measures ANOVA, ^*^p < 0.05, ^*^^*^p < 0.01, ^****^p < 0.0001, *n* = 12–15.

First, an open field (OF) test was performed to evaluate the locomotion and exploratory activity in the different genotypes (Figure 3). No genotype differences in the distance moved and velocity were observed within the same age between EN1-CB1-KO and EN1-CB1-WT (Figures 3A,C), and between EN1-CRE and EN1-WT (Figures 3B,D). However, the time spent in the center as a measure of anxiolytic-like behavior seemed to be increased in old EN1-CB1-KO mice as compared to EN1-CB1-WT controls [*t*_(27)_ = 2.903, *p* = 0.0073; Figure 3E], whereas anxiety-like behavior in young EN1-CB1-KO mice and EN1-CB1-WT controls did not differ (Figure 3E). To exclude any effect of the EN1-CRE transgene, we observed no differences in both young and old EN1-CRE mice as compared to the respective WT controls (Figure 3F). However, if we compare the 12 weeks old EN1-CB1-WT controls to the 30 weeks old WT controls (Figure 3E), old EN1-CB1-WT mice are more anxious than young EN1-CB1-WT mice (Figure 3E; *p* < 0.05), thus, anxiety-like behavior increases with the age in WT animals, which is consistent with a previous study (Li et al., 2020), whereas this age effect is not observed in the old EN1-CB1-KO mice leading to decreased anxiety-like behavior of old mutant mice as compared to their respective WT controls. As an age effect, the moved distance and the velocity decreased in old as compared to young mice in both lines without genotype differences (Figures 3A–D; *p* < 0.0001). Lastly and importantly, no differences in distance moved and velocity were observed within each mouse line (EN1-CB1-KO and EN1-CRE lines compared to EN1-CB1-WT and EN1-WT, respectively), and the age difference was seen in both lines. This indicates that no alterations in locomotion occurred due to the loss of CB1 (Figures 3A,C) or one En1 allele (Figures 3B,D), also consistent with our histological analyses detecting no CB1 expression in the SNc.

The accelerating rotarod was used to analyze motor coordination and sensorimotor learning (Figures 3G–J). Mice were trained in three consecutive days with three trials on each day. The overall behavior of the different genotypes was not different as analyzed by repeated measures ANOVA (*p* > 0.05). Thus, deletion of one En1 allele or two CB1 alleles did not impair motor coordination and balance. Furthermore, sensorimotor learning was also unaffected, as all mice showed similar improvements in latency to fall off the rod. In contrast to the decreased locomotion activity in old mice observed in the OF, old mice did not display a decreased motor coordination and balance compared to young mice.

#### 3.3.2 Anxiety-like behavior evaluated in the light/dark avoidance test

**Figure 4:**
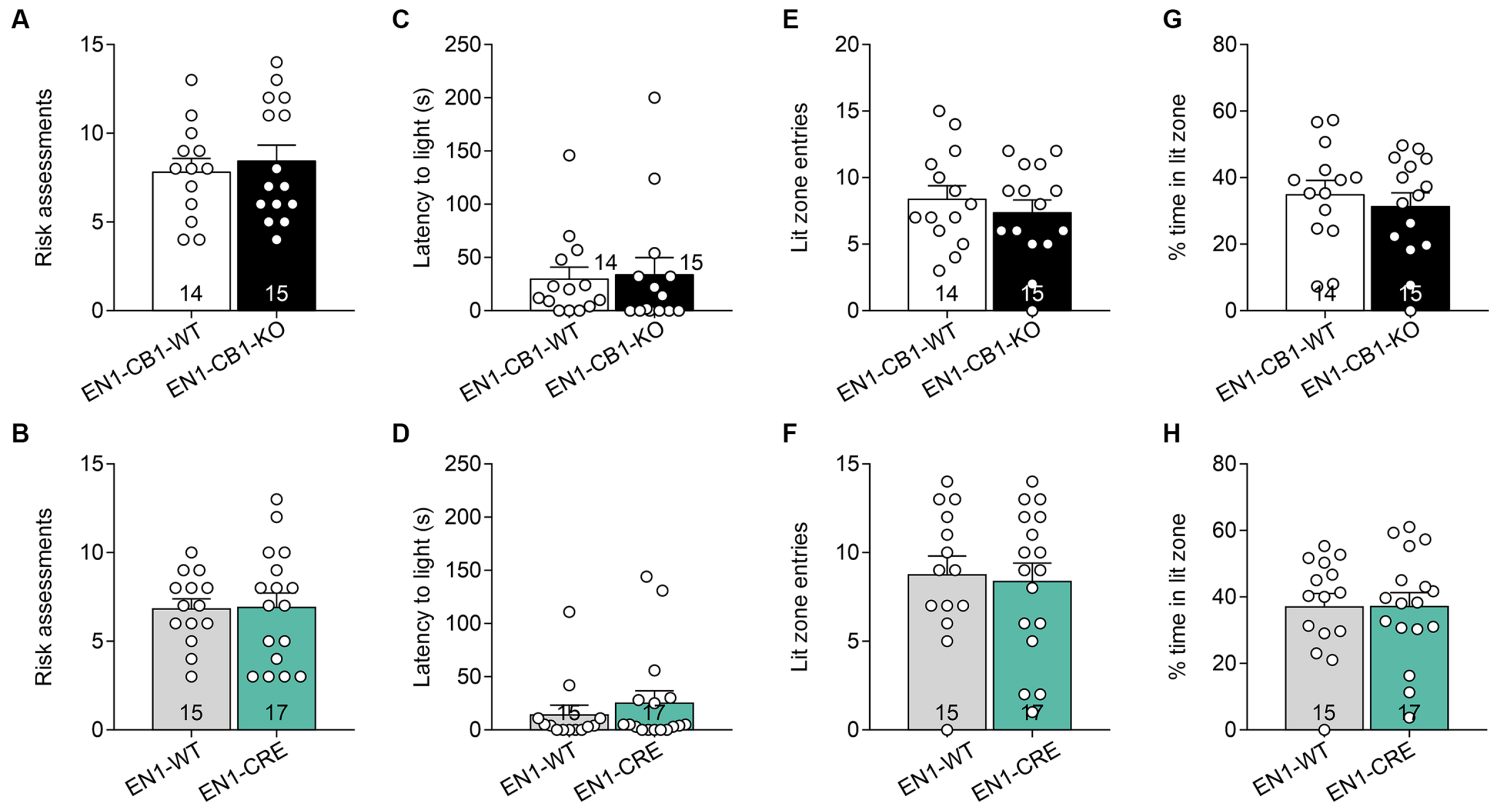
CB1 deletion in En1-expressing cells did not impair anxiety-like behavior measured by the light/dark test. Analysis of the LD test revealed no differences in EN1-CB1-KO **(A,C,E,G)** and EN1-CRE **(B,D,F,H)** mice (12–15 weeks old) and their respective controls concerning the following parameters: **(A,B)** risk assessments. **(C,D)** Latency to enter light zone. **(E,F)** Lit zone entries. **(G,H)** Time spent in the lit zone (%). Data are expressed as mean ± SEM for each group. Two-tailed unpaired Student’s *t*-test, *n* = 14–17.

The light/dark avoidance (LD) test measured anxiety-like behavior in EN1-CB1-KO and EN1-CRE lines based on the natural aversion to illuminated areas and spontaneous exploratory behavior in novel environments. Mice (12–15 weeks old) were placed in the dark compartment for 30 s to habituate until the entry site was opened and were then allowed to freely explore the entire box, consisting of a lit and dark compartment, for 5 min. Risk assessment behavior at the opening between the two compartments and latency of the first entry to the lit compartment were scored. Furthermore, the number of entries to the light and the time spent in the lit zone were used to determine aversive behavior. Analysis of the LD test revealed no differences in EN1-CB1-KO (Figures 4A,C,E,G) and EN1-CRE mice (Figures 4B,D,F,H), as compared to their corresponding littermates in the respective parameters, suggesting no changes in anxiety-like behavior at this age.

#### 3.3.3 Despair behavior in an inevitable situation

**Figure 5:**
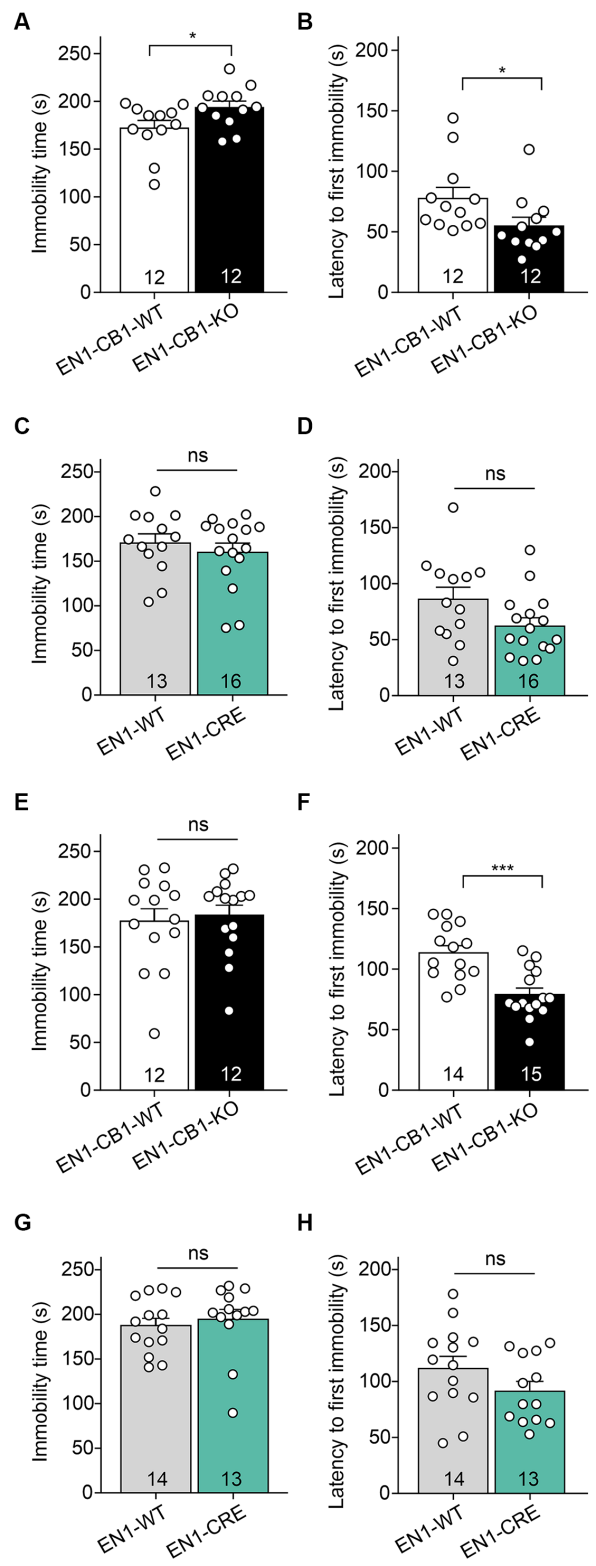
EN1-CB1-KO mice display an increased depressive-like behavior in the forced swim test. In **(A–D)** the analysis of 14 weeks old mice is shown, in **(E–H)** of 30–31 weeks old mice. EN1-CB1-KO mice spent more time immobile **(A)** and showed shorter latency to the first immobility **(B)** than EN1-CB1-WT littermate controls. No differences were observed between EN1-CRE and EN1-WT littermates in immobility time **(C)** and latency to the first immobility **(D)**. The significant effect in the FST in EN1-CB1-KO mice could also be observed in older mice in the latency to the first immobility compared to their WT litter mates **(F)**, whereas EN1-CRE and EN1-WT showed no difference **(H)**. However, the immobility time revealed no differences in both lines **(E,G)**. Data are expressed as mean ± SEM for each group. Two-tailed unpaired Student’s *t*-test, ^*^p < 0.05, *n* = 12–16 **(A–D)**, ^***^p < 0.001, *n* = 12–15 **(E–H)**.

Despair behavior (also considered as depressive-like behavior) was assessed in the forced swim test (FST). EN1-CB1-KO mice and EN1-CB1-WT littermates at 12–15 weeks of age were subjected to the test, which employs forced swimming to induce passive emotional coping characterized by increased time spent in immobility posture. EN1-CB1-KO mutant mice showed significantly increased immobility time [*t*_(22)_ = 2.181, *p* = 0.0401; Figure 5A] and decreased latency to the first immobility [*t*_(22)_ = 2.082, *p* = 0.0492; Figure 5B] as compared to controls, suggesting a depressive-like behavior. In the EN1-CRE control line, no differences between genotypes were detected, neither in the immobility time [*t*_(27)_ = 0.7466, *p* = 0.4618; Figure 5C] nor in the latency to the first immobility time [*t*_(27)_ = 1.999, *p* = 0.0557; Figure 5D], indicating that the inactivation of one En1 allele does not affect despair behavior. Solely, the conditional CB1 deletion in En1-positive cells leads to an increased depressive-like behavior. We performed the FST also with 30–31 weeks old mice (Figures 5E–H). A significant effect in the decrease of latency to the first immobility could also be observed in old EN1-CB1-KO mice [*t*_(27)_ = 4.293, *p* = 0.0002] as compared to controls (Figure 5F), whereas EN1-CRE and EN1-WT animals showed no difference (Figure 5H). The immobility time however revealed no difference neither in the EN1-CB1-KO line nor in the EN1-CRE line (Figures 5E,G).

#### 3.3.4 Sucrose preference test to address anhedonia

**Figure 6:**
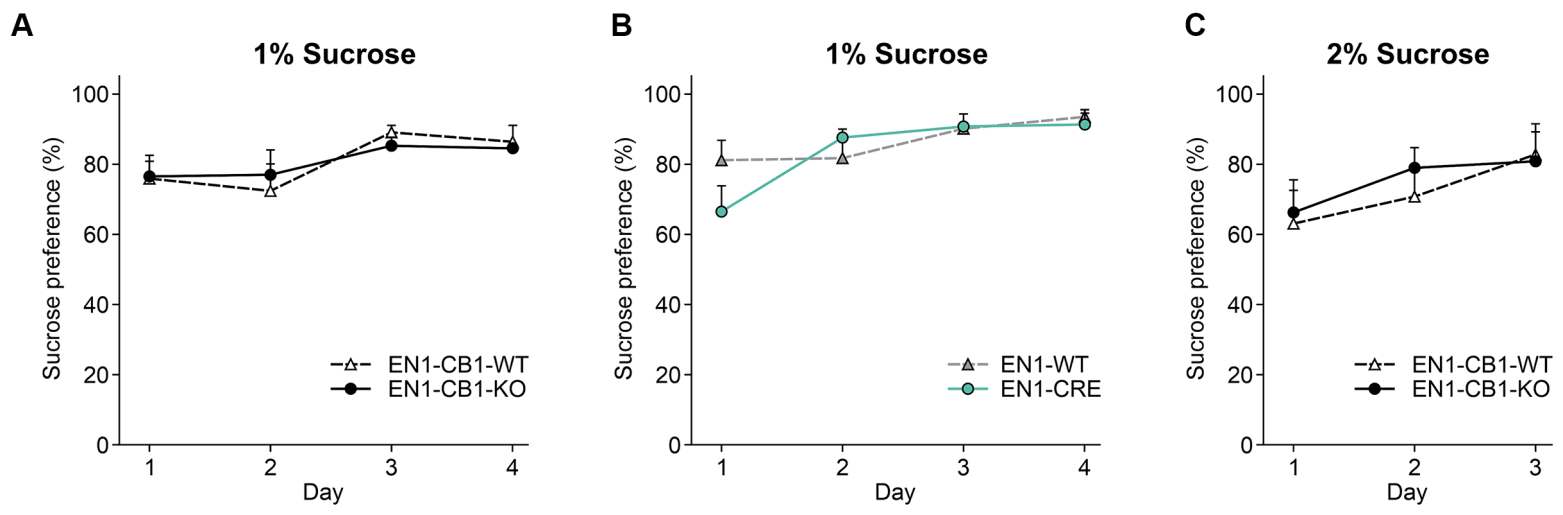
CB1 deletion in EN1-positive cells did not affect anhedonia. EN1-CB1-KO mice (*n* = 14) and EN1-CB1-WT littermates (*n* = 11), at the age of 13–17 weeks, showed a strong preference for sucrose (1%) within 4 days of testing without a difference between genotypes **(A)**. Although EN1-CRE mice (*n* = 11) displayed a tendency to decreased preference for 1% sucrose on day 1 compared to EN1-WT controls (*n* = 11), they showed the same preference on the following days **(B)**. A second group of EN1-CB1-KO mice (*n* = 15) was tested for 3 days with a 2% sucrose solution. No differences between genotypes (EN1-CB1-KO mice and littermates) were observed **(C)**. Data are expressed as mean ± SEM for each group. ANOVA with repeated measures.

Mice usually show a strong preference for sucrose, and reduced consumption is denoted as a depressive-like behavior in terms of anhedonia. Therefore, a sucrose preference test (SPT) was conducted with 13–17 weeks old mice using a two-bottle free-choice paradigm to evaluate genotype differences in sucrose-sweetened water drinking behavior. Sucrose preference was calculated as a percentage of the volume of sucrose intake over the total volume of fluid intake. All genotypes showed a preference for sucrose and increased their sucrose consumption over time. EN1-CB1-KO mice showed a similar preference for sucrose of 76.85% ± 4.5 compared to EN1-CB1-WT littermates (76.2% ± 6.8) on the first day of testing (Figure 6A). On day 3, they showed a preference of 85.7% ± 6.0 (EN1-CB1-KO) and 89.6% ± 2.1 (EN1-CB1-WT). No significant genotype effect was revealed in the EN1-CRE line (*p* > 0.05 Figure 6B). Since we did not see a difference with 1% sucrose, we additionally compared the preference of a 2% sucrose solution between EN1-CB1-KO (*n* = 15) and EN1-CB1-WT mice (*n* = 15) over 3 days. We could not reveal any differences between genotypes, whereas the overall preference was slightly below the one for 1% sucrose for both, KO and WT animals (Figure 6C). Furthermore, it is important to note that the total intake of liquid (water and sucrose solution) did not differ between groups throughout the experiment (*p* > 0.05; data not shown).

#### 3.3.5 Food-related motivation with food restriction

**Figure 7:**
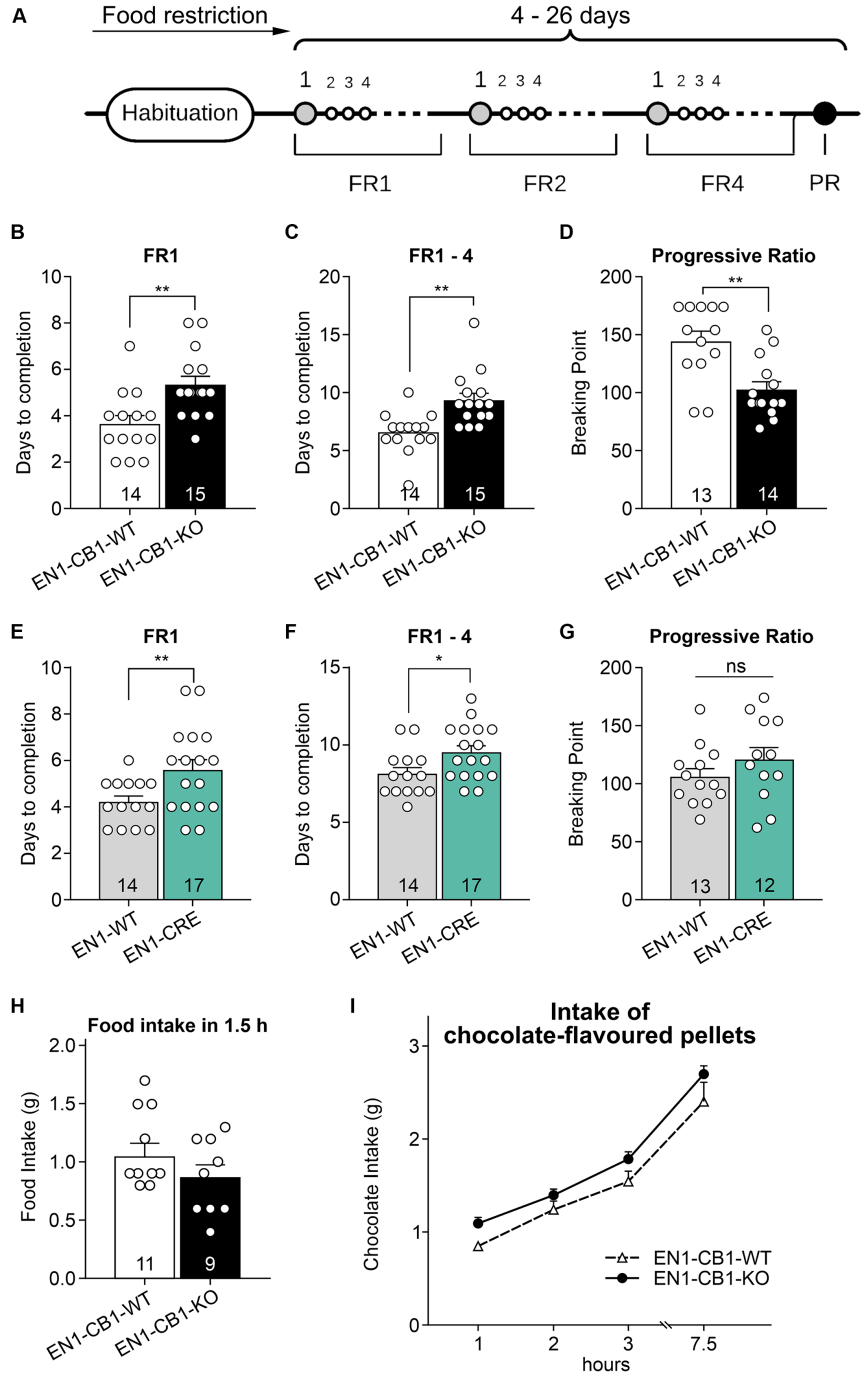
EN1-CB1-KO mice needed more days to accomplish fixed ratio training sessions for pellet self-administration and showed a decreased motivation in the progressive ratio test. **(A)** Timeline of the different phases during operant conditioning. After habituation and food restriction until mice (15–20 weeks old) lost 15% of their free-feeding body weight, they were trained daily in 30 min sessions under FR1, FR2, and FR4, followed by one 90 min PR session. The criterion for completion of the phases of the operant conditioning was acquired when mice consumed at least 50 pellets within one session. Every mouse was evaluated individually. In FR1, EN1-CB1-KO mice (*n* = 15) needed approximately 2 days more than EN1-CB1-WT control littermates (*n* = 14) to reach the criterion for entering FR2 **(B)** similar to EN1-CRE mice (*n* = 17) compared to EN1-WT controls (*n* = 14) **(E)**. For the entire training phase, including FR1, FR2, and FR4, EN1-CB1-KO mice even needed 2.7 days **(C)** and EN1-CRE mice 1.5 days more **(F)**. The mean BP measured in the PR session was significantly reduced in EN1-CB1-KO mice (*n* = 14) with 103 nose responses compared to EN1-CB1-WT littermates (*n* = 13), achieving 144 nose responses **(D)**. In the EN1-CRE mouse line, the BP was similar in both genotypes **(G)**. **(H,I)** To exclude underlying differences in hunger or chocolate taste preference, the food and chocolate intake was examined after the PR schedule. To ensure that motivation differences are not caused by an altered feeling of hunger, food intake of normal food pellets was measured after 12 h food deprivation **(H)** and independently also chocolate intake in a separate test **(I)**. The tests revealed that EN1-CB1-KO and EN1-CB1-WT mice did not differ in this regard. Data are expressed as mean ± SEM. ^*^p < 0.05, ^**^p < 0.01, *n* = 12–17 in FR and PR; food intake, *n* = 9–11; intake of chocolate-flavored pellets, *n* = 12–13. PR, progressive ratio; FR, fixed ratio; BP, breaking point.

To address a potential effect of CB1 ablation in En1 expressing mesDA neurons on motivation for highly palatable food, we examined motivation in food-restricted mice (15–20 weeks old) to consume chocolate-flavored pellets in a self-administration paradigm. EN1-CB1-KO (*n* = 15) and EN1-CB1-WT mice (*n* = 14) were trained under a fixed ratio (FR) 1 schedule of reinforcement followed by FR2 and FR4 to obtain chocolate-flavored pellets as reinforcers (Figure 7A). In FR1, EN1-CB1-KO mice needed approximately 2 days more to achieve 50 pellets within 30 min (EN1-CB1-KO: 5.3 ± 0.4 days; EN1-CB1-WT: 3.6 ± 0.4 days; Figure 7B) than EN1-CB1-WT control littermates, an observation which also persisted for the following FR schedules. For the entire training phase, including FR1, FR2, and FR4, EN1-CB1-KO mice needed 9.3 ± 0.6 days, while EN1-CB1-WT controls already accomplished these three phases after 6.6 ± 0.5 days (Figure 7C). Student’s *t*-test revealed significant differences between EN1-CB1-KO mice and the control group both in FR1 [*t*_(27)_ = 3.2, *p* = 0.0035] and the entire FR training [*t*_(27)_ = 3.544, *p* = 0.0015]. The breaking point (BP), referring to the maximal effort of nose responses an animal is willing to give to earn one pellet, was ascertained in the progressive ratio (PR) session. The cut-off was defined after 1.5 h or 29 collected pellets. BP values of EN1-CB1-KO mice were significantly reduced with only 103 nose responses compared to EN1-CB1-WT littermates achieving 144 nose responses after 1.5 h [*t*_(25)_ = 3.669, *p* = 0.0012; Figure 7D].

EN1-CRE mice (*n* = 17) also significantly differed from EN1-WT control littermates (*n* = 14) in the FR experiments (Figures 7E,F). Mutant animals needed approximately 2 days more under FR1 schedule [*t*_(29)_ = 2.514, *p* = 0.0177] and 1.5 days more for the entire training phase than EN1-WT controls [EN1-CRE: 9.5 ± 0.4; EN1-WT: 8.14 ± 0.4; *t*_(29)_ = 2.314; *p* = 0.0280]. However, the BP was not significantly different between both groups (Figure 7G).

Taken together, mutant mice with a deletion of one En1 allele, both in the EN1-CB1-KO and the EN1-CRE mouse line, therefore, needed more time in the training phase. Notably, however, the motivation measured by the BP in the PR session was altered only in the EN1-CB1-KO mouse line compared to EN1-CB1-WT littermates, but not in EN1-CRE mice and controls. Thus, the motivation is affected by the CB1 deletion in En1-positive cells, e.g., in the mesolimbic system, as EN1-CB1-KO mice showed less motivation in the PR as measured by the BP.

To ensure that the decreased motivation is independent of genotype-specific hunger levels, we measured at the time point of 1.5 h of feeding the amount of normal food intake in 12 h food-deprived EN1-CB1-KO mice and WT controls (after the PR schedule) (Figure 7H). Furthermore, we analyzed the craving for chocolate-flavored pellets. Therefore, mice were food-deprived for 12 h several days after the PR schedule in an independent test, before they were allowed to consume palatable chocolate pellets *ad libitum* for 7.5 h. Within this 7.5 h period the food intake was calculated at 1, 2, 3, and 7.5 h. Mice showed the same hunger levels and desire for chocolate-flavored pellets, regardless of the genotype (Figure 7I).

#### 3.3.6 Food-related motivation without food restriction

**Figure 8:**
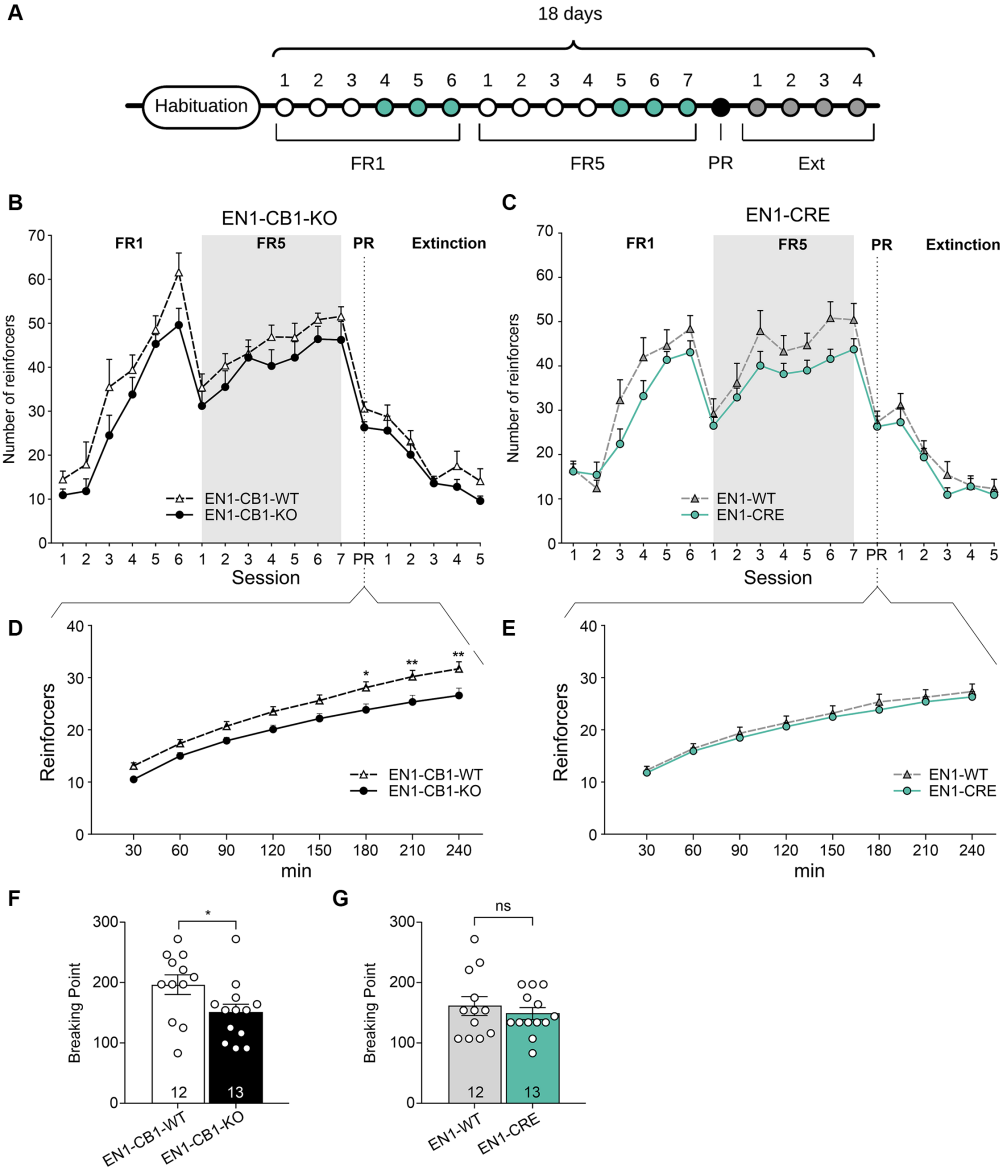
EN1-CB1-KO mice displayed a decreased chocolate-flavored pellet self-administration in the progressive ratio test. **(A)** Timeline showing the number of days of the different phases during operant conditioning and extinction. After habituation of 12 days, mice (38–44 weeks old) were trained daily in 1 h sessions for 6 days under FR1 and 7 days under FR5, followed by the 4 h PR session and extinction phase for 4 days. During the last three consecutive days of FR1 and FR5 (green), mice maintained a stable response with less than 20% deviation from the mean of the total number of reinforcers, which was the criterion for entering the next phase. **(B,C)** Overview graph of the mean number of reinforcers obtained in operant learning (1 h/day), the PR (4 h), and extinction sessions (1 h/day) by EN1-CB1-KO **(B)** and EN1-CRE **(C)** mice compared to their littermate controls. Mice were trained daily under an FR1 and FR5 schedule. After the 4 h PR session, the extinction phase was conducted. After extinction, mice were no longer reinforced after touch responses for chocolate-flavored pellets as they achieved the same number of reinforcers compared to the start of operant training. Mice did not show different levels of acquisition in operant conditioning learning and extinction of food self-administration. **(D,E)** EN1-CB1-KO mutants showed fewer reinforcers than controls **(D)**, whereas EN1-CRE did not differ from littermate controls **(E)**. Thus, the BP values of EN1-CB1-KO mice were also decreased compared to EN1-CB1-WT controls **(F)**, whereas EN1-CRE mice and EN1-WT control littermates displayed similar BP values **(G)**. Error bars represent ± SEM. ^*^p < 0.05; ^**^p < 0.01, EN1-CB1-KO: *n* = 13 in FR1-4 and PR; *n* = 8 during extinction; EN1-CB1-WT: *n* = 12 in FR1-4 and PR; *n* = 7–8 during extinction; EN1-CRE: *n* = 13 in FR1-4 and PR; *n* = 8 during extinction; EN1-WT: *n* = 12 in FR1-4 and PR; *n* = 7 during extinction. PR, progressive ratio; FR, fixed ratio; BP, breaking point.

Since food restriction is considered stressful in mice and the endocannabinoid signaling system is involved in stress coping, EN1-CB1-KO mice were tested for motivation for palatable chocolate-flavored pellets in a self-administration paradigm without food restriction. All mice were 38–44 weeks old. Moreover, sessions were performed during the dark phase using a reversed light/dark cycle. EN1-CB1-KO (*n* = 13) and EN1-CB1-WT mice (*n* = 12) plus EN1-CRE (*n* = 13), and EN1-WT (*n* = 12) animals were trained under an FR1 schedule during six sessions followed by seven sessions under FR5 to obtain chocolate-flavored pellets as reinforcers (Figure 8A).

Both lines (EN1-CB1-KO and EN1-CRE) increased reinforcers across FR1 and FR5 training phases. Mice acquired operant responses similarly without significant differences, although there was an overall tendency of a decreased acquisition of mutants compared to controls in both mouse lines. Both EN1-CB1-KO and EN1-CRE mice seem to require more time to acquire operant conditioning learning than EN1-CB1-WT or EN1-WT littermate controls (Figures 8B,C). However, in contrast to the PR schedule with food restriction, where the Student’s *t*-test reveals significant differences between both mutants and their control groups during the entire FR training, differences did not gain significance in this schedule for both mouse lines, indicating similar acquisition levels in the operant conditioning learning.

During the PR session, the initial tendency of a decreased reinforcer achievement for EN1-CB1-KO mice compared to EN1-CB1-WT littermates became statistically significant starting at 180 min, as revealed by two-way ANOVA [*F*_(1, 20)_ = 8.173, *p* = 0.0097; Figure 8D]. Accordingly, BP values were also significantly decreased in EN1-CB1-KO mice compared to EN1-CB1-WT controls [*t*(23) = 2.187, *p* = 0.0392; Figure 8F]. On the contrary, EN1-CRE mice and EN1-WT control littermates displayed the same motivation for chocolate-flavored pellets in the entire PR session (Figures 8E,G).

Thus, the decreased motivation is solely attributed to the CB1 deletion and not to the impairment of one En1 allele. Moreover, the decreased motivation of EN1-CB1-KO mice observed in the PR paradigm using food restriction (Figure 7) could be confirmed. In conclusion, the En1-specific deletion of CB1 in the mesolimbic systems caused a decreased motivation for highly palatable food.

After the PR session, a subset of mice of both lines (EN1-CB1-KO *n* = 8; EN1-CB1-WT *n* = 7; EN1-CRE *n* = 8; EN1-WT *n* = 7) was trained to extinguish operant responses to palatable chocolate food (Figures 8B,C). The extinction criterion was achieved within 5 days when mice gave the same number of touch responses as at the beginning of the self-administration paradigm. During extinction, no genotype differences were found. These data indicate that mutants and controls learned to the same extent that the light stimulus is no longer followed by positive reinforcement.

## 4 Discussion

In the present study, we validated a new genetic mouse line providing an excellent tool to examine the neurobiological function of CB1 in mesDA neurons of the VTA and SNc. Our goal was to inactivate CB1 expression in cells expressing En1, beginning from mesDA neuron differentiation in the isthmic organizer. Thus, CB1 receptor was genetically deleted from a very early time point of mesDA neuron development.

Our work on adult animals indicates that CB1 is expressed in a subset of dopaminergic neurons of the VTA and shows that CB1 deletion in mesDA neurons selectively elevates depressive-like behavior: EN1-CB1-KO mice show an increased immobility time in young mice and shortened latency to the first immobility in young and old mice in the forced swim test. Furthermore, CB1 deletion attenuates motivation to consume palatable food, i.e., the maximal effort exerted to obtain access to chocolate pellets was significantly reduced in EN1-CB1-KO mice, as measured by responding under a PR schedule. But CB1 deficiency had no impact on responsiveness to sucrose. Moreover, the behavioral analyses revealed that locomotion was not affected, and in anxiety-like behavior that old EN1-CB1-KO mice did not show age related increase in anxiety, as their WT counterparts did.

In summary, our data suggest that CB1 directly modifies the mesocorticolimbic pathway implicated in despair behavior and motivation. In contrast, the nigrostriatal pathway controlling voluntary movement is unaffected, which is in agreement with the observation of CB1 being not expressed in SNc, and this observation is in accordance with a recent work (Han et al., 2023).

### 4.1 Stereological counts of mesencephalic dopaminergic neurons

We first evaluated the number of mesDA neurons in the VTA and SNc of 42 weeks old mice of the EN1-CB1-KO and EN1-CRE mouse lines. Stereological counts revealed no reduction of the total number of TH-positive cells neither in the VTA nor in the SNc of both lines. Hence, we conclude that neither the heterozygosity of the En1 gene nor the ablation of CB1 caused degeneration of mesDA neurons in adult mice. This was an important finding in the light that two studies previously showed that replacing the first 111 amino acids of one En1 allele with the lacZ gene leads to the degeneration of mesDA neurons in the SNc and the VTA (Sonnier et al., 2007; Nordström et al., 2015). However, our results agree with Sgadò et al. (2006) previous report, demonstrating that En1 heterozygous mice display an unchanged number of mesDA neurons in the VTA as compared to wild-type controls. Sonnier et al. (2007) argued that different genetic backgrounds might cause these discrepancies, as they used Swiss mice (Janvier, Le Genest-St.-Isle, France), and Sgadò et al. (2006) used C57BL/6 mice as we did in this study. Nordström et al. (2015) analyzed the mouse line of Sonnier et al. (2007) but maintained on an OF1 genetic background, and the results agreed with the findings of Sonnier et al. (2007). Thus, we conclude that the replacement of the first 111 amino acids of one En1 allele does not lead to a degeneration of mesDA neurons in mice *per se*, but degeneration might depend on the genetic background. Another possible explanation might be that the integrated gene is responsible for the degeneration of the mesDA neuron population. While the lacZ gene insertion in the En1 locus leads to a progressive decline of mesDA neurons (Sonnier et al., 2007; Nordström et al., 2015), the tau-LacZ (Sgadò et al., 2006) (its protein being not in the cell soma but mostly in axons) and Cre genes do not lead to a cell loss of VTA neurons. Yet, the expression of the lacZ-sequence is a widely used reporter tool to assess the expression of genes of interest in mice. However, once LacZ is activated, it is continuously expressed, leading to protein accumulation representing one of the hallmarks of neurodegenerative diseases (Braak and Braak, 1991; Kobayashi and Chen, 2005). LacZ expression in glutamatergic neurons in the cortex, for example, causes substantial deficits in hippocampus-dependent memory, as well as structural changes of, e.g., dendrite morphology and reduction of hippocampal volume. GFP expression in the same cell populations, on the other hand, does not result in deficiencies in cognition or structure (Reichel et al., 2016). Taken together, the findings of Reichel et al. (2016) might explain the effect of DA cell loss in En1^lacZ/+^ mice, which is not present in the En1^Cre/+^ lines.

### 4.2 CB1 receptor expression in mesencephalic dopaminergic neurons

In the present study, we identified CB1 in mesDA neurons in adult brain. Whereas no CB1 was observed in the SNc, neurons in the VTA co-expressing CB1 and TH (a marker for mesDA neurons in the ventral tegmentum) were detected. Consequently, in mesDA neurons of EN1-CB1-KO mutant mice, CB1 mRNA expression is lost. Given that CB1 protein, e.g., detected by ligand binding, or CB1 mRNA levels in mesDA neurons had been widely reported to be absent or their presence has been controversially discussed, it has been assumed that a direct effect of the ECS on DA cells seemed unlikely (Melis et al., 2012; Wenzel and Cheer, 2018). Thus, CB1 might indirectly modulate DA neurons via excitatory and inhibitory mechanisms of surrounding GABAergic neurons and glutamatergic long-range inputs. However, by going through studies, *in situ* hybridization, binding, and immunohistochemistry experiments from the 1990s and 2000s revealed both expression and localization of CB1 receptor in mesDA neurons. Hence, our findings agree with the study by Matsuda et al. (1993), reporting also low and scattered CB1 mRNA hybridization signals throughout the rat VTA. Furthermore, the missing CB1 expression in the SNc is in line with a previously described study in rat (Julian et al., 2003). Recently, another study showed CB1 expression in the mouse VTA and very low in the SNc using RNAscope (Han et al., 2023). Considering that the VTA dopamine-releasing neurons are heterogeneous in their afferent and efferent connectivity and, in some cases, release GABA or glutamate in addition to dopamine, no studies to date have demonstrated the direct function of CB1 receptor in a specific subset of VTA mesDA neurons. Interestingly, we identified most co-expressing neurons in the lateral VTA, comprising the parabrachial pigmented nucleus and paranigral nucleus of the VTA (pnVTA). Increasing evidence suggests heterogeneity in neuronal subtypes and anatomical localization in the VTA, including transmitter and neuropeptide systems that may modulate dopaminergic outputs (Jhou et al., 2009; Tan et al., 2012; Van Zessen et al., 2012; Morales and Margolis, 2017). However, the exact processes and regulatory pathways are still an unexplored area. Interestingly, neuropeptide nociceptin-expressing neurons were revealed in the paranigral nucleus (pnVTA) that, in turn, project to mesDA neurons in the VTA – a neurocircuit negatively regulating motivation and reward-seeking in mice (Parker et al., 2019). To date, we did not analyze whether CB1 expressing cells also express nociceptin, but we found in the supplementary data list (Parker et al., 2019) prepronociceptin and CB1 mRNA expressed in the pnVTA neurons (see below).

Collectively, our data support a unique role of the ECS in the mesolimbic system. While in most literature, a direct effect of the ECS on DA system in the VTA and SNc is assumed to be absent and research focuses on the GABAergic and glutamatergic afferents, we assume that it will be of great interest to determine whether CB1 acts also directly in mesDA neurons, in particular in the VTA. The ECS in the dopaminergic system could be responsible to withdraw the inhibition of reward seeking behavior conducted by stimulated nociceptin expressing pnVTA neurons, when endocannabinoids from DA neurons cause a retrograde inhibition upon stimulation by pnVTA neurons, which leads to a withdrawal of inhibiting the motivation and thereby a dampening of negatively controlling an exaggerating motivation. In EN1-CB1-KO mice, the suppression of inhibition is missing and the lower reward-seeking behavior is prominent. This could be an explanation for our results in the progressive ratio task. In fact, to corroborate the possibility of this mode of functioning, we found in the TRAP RNAseq data list that in pnVTA cells, besides prepronociceptin, also En1, TH and CB1 is expressed (Gene counts table from GEO, Accession: GSE 108813) (Parker et al., 2019). Thus, En1-Cre in these nociceptin positive neurons could delete CB1 expression, and the retrograde inhibition through CB1 on these motivation inhibiting neurons cannot take place in EN1-CB1 mutants, and for this reason they show less motivation.

### 4.3 Locomotion behavioral analyses and anxiety-like behavior

Considering our histological data indicating CB1 loss in the ventral midbrain, we were interested whether the CB1 deletion in this area and in precursors of mesDA neurons leads to alterations in the behavior of adult EN1-CB1-KO mice. Therefore, we decided to investigate test paradigms addressing the nigrostriatal and mesolimbic systems. Impairments of the nigrostriatal pathway lead to motor dysfunctions, whereas the mesolimbic system is involved in motivation, reward, and addiction. Although we could not find a decline of mesDA neurons in adult mice, we performed all behavior tests with both mouse lines, EN1-CB1-KO and EN1-CRE, to exclude a behavioral phenotype due to the impairment of one En1 allele.

Concurrent with the histological outcomes, we could not find any differences in motor performance, as mice of both lines performed similarly in the OF and in the accelerating rotarod test. Furthermore, we could exclude a genotype-dependent decline of locomotion in older mice by OF and rotarod tests. Thus, neither the deletion of one En1 allele nor the CB1 ablation impairs locomotion or sensorimotor learning in young and old mice. In the OF test, the age-related increase in anxiety-like behavior, measured as decreased time spent in the center was observed in EN1-CB1-WT at 30 weeks of age as compared to 12 weeks of age. This is congruent with the work by Li et al. (2020), who reported increased anxiety-like behavior in 12 months old C57BL/6 J mice as compared to mice at 2 months of age. The age related increase of anxiety appears not to be present in 30 weeks old EN1-CB1-KO mice, nor is it apparent in the EN1-CRE line. At this point, we are not able to explain this phenotype, nor can we explain why it is not observed in the EN1-WT mice. In addition, there was no difference seen in the LD test regarding risk assessment behavior based on avoidance of light illuminated area. Thus, further investigations would be required to study in detail anxiety-like behavior upon CB1 loss in EN1-positive cells.

In the accelerating rotarod test mice improved in motor coordination, balance and sensorimotor learning without genotype differences and without showing an age effect. However, Sonnier et al. (2007) reported that En1^lacZ/+^ mice have a deficit in motor coordination and sensorimotor learning in the rotarod and show an abnormal spontaneous motor activity in the OF test. Instead, the EN1-CB1-KO mice used in our study did not show a phenotype in motor capabilities. The reason why the nigrostriatal pathway related functions are not affected, is not clear but is congruent with our expression studies showing no CB1 mRNA expression in SNc DA neurons.

### 4.4 Depressive-like behavior and anhedonia

Since several studies showed that the ECS is also involved in the modulation of depression (Patel and Hillard, 2009), we were moreover interested in the impact of the CB1 ablation in the VTA in this regard. To date, there are inconsistencies regarding cannabis use observed in the human population: the use of cannabis may lead to the onset of depression, but on the contrary, depression may also lead to the onset or increase in cannabis use frequency. However, there is preclinical evidence that alteration in the ECS could potentially benefit patients suffering from depression (Feingold and Weinstein, 2021), but cannabis abuse is often associated with depression and bipolar disorder (El-Alfy et al., 2010). Therefore, the role of CB1 still remains elusive and needs to be further investigated. To further address the role of CB1 in the mesolimbic system, we performed the FST, a test originally developed in rats to investigate the effects of anti-depressant drugs, whereby the drug-induced increase in struggling and decrease in floating in the inescapable water bath was considered as the positive therapeutic effect. The FST has been widely used in preclinical research, but covers only a subset of possible pathological changes described in human depression, i.e., despair behavior in an inescapable situation, and, therefore gives a limitation to this test. Despite this limitation, the FST in the analysis of mutant mice is useful and can give us information about genotype differences in despair behavior and coping strategy. The term “depressive-like” behavior is often used, but behaviorally intends to describe “despair” behavior. Interestingly, our data demonstrate that EN1-CB1-KO mice show increased immobility and decreased latency to the first immobility compared to control mice. Hence, we conclude that CB1 deletion in En1-expressing cells leads to depressive-like behavior congruent with the results of, e.g., full CB1-KO mice (Steiner et al., 2008b). In contrast, mice lacking CB1 in principal forebrain neurons and in GABAergic neurons, behave like wild-type controls (Steiner et al., 2008a). However, interestingly mice lacking CB1 in cortical glutamatergic neurons show less despair behavior (Steiner et al., 2008a). Because of the fact that both pro- and anti-depressant effects have been reported, and that both, agonists and antagonists of the cannabinoid receptors, can act similarly to anti-depressants (Gorzalka and Hill, 2011), it is important to understand the underlying mechanisms of these complex interactions.

Another hallmark of depression is altered responsiveness to rewarding stimuli, such as impaired hedonic motivation (Willner, 1997). There is solid research suggesting that the ability to process rewarding stimuli requires ECS activity. CB1 deletion or blockade can attenuate the motivational, appetitive, and reinforcing effects of a diversity of rewarding stimuli, such as sucrose (Arnone et al., 1997; Freedland et al., 2000; Sanchis-Segura et al., 2004). Thus, the deficiency of ECS signaling may cause anhedonia. In contrast to full CB1-KO mice showing reduced responsiveness to sucrose (Sanchis-Segura et al., 2004), our set of experiments observed similar sucrose consumption in the two-bottle choice test of mutant and control mice, suggesting no anhedonic phenotype of EN1-CB1-KO mice. Thus, these processes seem to be independent of CB1 receptor expression in En1-expressing DA cells.

### 4.5 Progressive ratio test

The ECS plays a critical role in central mechanisms controlling appetite and food reward. It is well-known that in humans, for instance, cannabis increases the consumption of palatable food (Abel, 1975). In Progressive Ratio (PR) paradigms, designed to evaluate food-related motivation, mice are offered a highly preferred reward that can only be obtained through the exertion of effort (Hodos, 1961). In our first PR paradigm, mice were food-deprived by reducing the initial body weight to 85% during the experiment to motivate animals participating in the task and thus, learning the nose poking response. As a result, we showed that the learning of operant conditioning maintained by chocolate-flavored pellets was impaired in EN1-CB1-KO and EN1-CRE mice as they both require more time to develop learning than their respective control littermates. Consequently, under food-deprived conditions, the deletion of one En1 allele has an impact on operant learning. Nevertheless, CB1 receptor in En1 expressing cells might also be implicated in conditioning learning, supported by the observation that the difference is even more prevalent in the EN1-CB1-KO mouse line.

While operant responding on an FR schedule is a powerful method to evaluate the ability to learn a response to obtain a reinforcer, it might not reflect the degree of motivation to work to obtain the reinforcer. Thus, we also analyzed the behavior in a PR reinforcement schedule, in which increasing responses are progressively required to obtain the reward. Indeed, EN1-CB1-KO mice showed a significantly reduced motivation compared to littermates, measured by the BP, whereas the motivation of EN1-CRE mice remained unaffected as compared to their control littermates. Hence, we can conclude that motivation decreases specifically by the CB1 ablation in mesDA neurons. To ensure that the decreased motivation is independent of differences in hunger levels triggered by the genotype, we furthermore measured the amount of food intake in food-deprived mice. Mice consumed the same amount of food, regardless of the genotype. However, since ECS signaling is critical for stress and food intake regulation (Matias et al., 2006; Steiner et al., 2008a; Kirkham, 2009), we conducted a second self-administration PR paradigm in which the mice were fed *ad libitum*. Indeed, the mice were not overfed, and operant training did not significantly modify body weight as it was maintained at least at about 95% of the initial body weight. Moreover, we switched the day/night cycles, and mice could perform in their active, i.e., dark phase. Interestingly, in contrast to the previous experimental conditions under food restriction, our data revealed no significant change in acquisition levels of operant learning in fixed ratio schedules between mutant and wildtype in both mouse lines. Furthermore, EN1-CB1-KO mice displayed a decreased reinforcer achievement compared to littermates during the PR session, comparable to food-restricted mice. In the EN1-CRE mouse line, no differences were obtained, congruent with food-restricted mice of the prior PR schedule. Thus, the decreased motivation seems to be solely attributed to the CB1 deletion. Moreover, we evaluated that food restriction was not necessary to produce cue-induced seeking in this mouse line.

It is interesting to note that the decreased breaking point number in the progressive ratio in EN1-CB1-KO as compared to EN1-CB1-WT resembles the pharmacological effect of the D2/D3 receptor antagonist raclopride, Heath et al. (2015) suggesting overlapping signaling pathways of these neuromodulatory systems in the regulation of reward behavior. Our findings also agree with a growing body of research demonstrating that the ECS is implicated in the regulation of positive reward and motivational aspects of highly palatable food (Rowland et al., 2001; Miller et al., 2004; Domingo-Rodriguez et al., 2020). Previous research suggests that exposure to highly palatable food and motivational attributes may induce the eCB tone in the limbic region (e.g., NAc), resulting in dopamine release and heightened rewarding effects after consumption. While THC has been shown to stimulate the intake of chocolate-containing food without having an impact on regular food intake (Koch and Matthews, 2001), the CB1 antagonist rimonabant reduces the consumption of a highly palatable chocolate-flavored drink and blocks palatable food-induced dopamine release in the NAc (Maccioni et al., 2008). Reports of full CB1-KO mice exhibiting reduced sensitivity to the motivating properties of food are in line with these studies as they show lower levels of responding for sweet food and achieving lower breaking points (Sanchis-Segura et al., 2004). Parker et al. (2019) reported a nociceptin circuit regulating motivation and reward-seeking in mice. As already mentioned, nociceptin is enriched in the pnVTA (pnVTA^Pnoc^ neurons), which projects to the lateral parts of the VTA. Using fiber photometry experiments, it has been shown that the pnVTA^Pnoc^ neurons are necessary to limit motivation to obtain rewards, and nociceptin and its receptor NOPR play a critical role in this process. While mice performed a PR test to obtain a sucrose reward, Parker et al. showed that the activity of pnVTA^Pnoc^ neurons was low when the reward was easy to obtain, whereas it increased with the number of nose pokes and was highest at the BP. Selective ablation, chemogenetic inhibition, and photoinhibition of pnVTA neurons showed that the number of nose pokes increased in the PR task. In contrast, photostimulation and chemogenetic stimulation of pnVTA^Pnoc^ neurons resulted in a reduction in the number of nose pokes and the rewards obtained during the PR task, which was again blocked by the administration of a NOPR antagonist. Administration of a selective NOPR agonist reduces the number of nose pokes in the PR task in wild-type mice but not in Nopr^−/−^ mice. Deletion of the receptor in mesDA neurons of the VTA resulted in an increased number of rewards in the PR task. In turn, selective reexpression of Nopr in neurons of Nopr−/− mice and administration of a NOPR agonist led to a lower number of rewards obtained in the PR task (Parker et al., 2019). CB1 might possibly be involved in the regulation of this system (see above).

In conclusion, our data demonstrate that the deletion of CB1 in En1-expressing cells was sufficient to reduce motivation for highly palatable food. Additionally, we showed that CB1 is not required for the extinction of the stimulus–response association in this appetitively motivated learning task, implying that distinct molecular pathways may govern the training, acquisition, and extinction phases. Future studies will be required to corroborate the function of CB1 in mesDA neurons and neural circuits implicated in food-related motivation.

## Data availability statement

The original contributions presented in the study are included in the article/supplementary material, further inquiries can be directed to the corresponding author.

## Ethics statement

The animal study was approved by Landesuntersuchungsamt Koblenz, Rhineland-Palatinate, Germany. The study was conducted in accordance with the local legislation and institutional requirements.

## Author contributions

SB: Formal analysis, Investigation, Methodology, Writing – original draft. BL: Conceptualization, Data curation, Funding acquisition, Resources, Writing – review & editing. CH: Conceptualization, Data curation, Investigation, Project administration, Supervision, Writing – original draft, Writing – review & editing.
